# Spiking Recurrent Neural Networks Represent Task-Relevant Neural Sequences in Rule-Dependent Computation

**DOI:** 10.1007/s12559-022-09994-2

**Published:** 2022-02-05

**Authors:** Xiaohe Xue, Ralf D. Wimmer, Michael M. Halassa, Zhe Sage Chen

**Affiliations:** 1 Courant Institute of Mathematical Sciences, New York University, New York, NY, USA; 2 Department of Brain and Cognitive Sciences, Massachusetts Institute of Technology, Cambridge, MA, USA; 3 Department of Psychiatry, New York University School of Medicine, New York, NY, USA; 4 Department of Neuroscience and Physiology, New York University School of Medicine, New York, NY, USA; 5 Neuroscience Institute, New York University School of Medicine, New York, NY, USA

**Keywords:** Prefrontal cortex, Spiking recurrent neural network, Neural sequence, Neural oscillations

## Abstract

**Background:**

Prefrontal cortical neurons play essential roles in performing rule-dependent tasks and working memory-based decision making.

**Methods:**

Motivated by PFG recordings of task-performing mice, we developed an excitatory-inhibitory spiking recurrent neural network (SRNN) to perform a rule-dependent two-alternative forced choice (2AFC) task. We imposed several important biological constraints onto the SRNN, and adapted spike frequency adaptation (SFA) and SuperSpike gradient methods to train the SRNN efficiently.

**Results:**

The trained SRNN produced emergent rule-specific tunings in single-unit representations, showing rule-dependent population dynamics that resembled experimentally observed data. Under varying test conditions, we manipulated the SRNN parameters or configuration in computer simulations, and we investigated the impacts of rule-coding error, delay duration, recurrent weight connectivity and sparsity, and excitation/inhibition (E/I) balance on both task performance and neural representations.

**Conclusions:**

Overall, our modeling study provides a computational framework to understand neuronal representations at a fine timescale during working memory and cognitive control, and provides new experimentally testable hypotheses in future experiments.

## INTRODUCTION

A biological brain consists of large-scale neuronal networks with recurrent connections that perform computations in complex task behaviors. In recent years, recurrent neural networks (RNNs) have been widely used for modeling a wide range of neural circuits, such as the prefrontal cortex (PFC), parietal cortex, and primary motor cortex (M1), in performing various cognitive and motor tasks [1–9]. Varying types of assumptions have been made in different computational models: leaky integrate-and-fire (LIF) vs. conductance-based compartment model, rate vs. spiking-based model. Spikes are the fundamental language that the brain uses to represent and communicate information. Inspired by information processing in the brain, spiking neural networks (SNNs) attempt to mimic the mechanistic operation and computation among spiking neurons [10]. Although SNNs are derived from biologically inspired neurons, neural plasticity or learning for SNNs have been less efficient, (i.e., slow convergence) compared to the rate-based models [11–14]. In recent years, researchers have developed various methods for improving the learning speed of feedforward and recurrent SNNs [15–18].

Working memory and attention control are two fundamental functions in cognition and decision making. The two-alternative forced choice (2AFC) task is a classic experiment in psychophysics and electrophysiology to measure the working memory and cognitive control capacity. Recent experiments [19,20] have shown that prelimbic PFC neurons in task-performing mice demonstrate rule-specific and timing-specific neuronal responses during the delay (i.e., working memory) period, displaying emergent rule-specific neural sequences. Sequentially activated neuronal activities (“neural sequences”) have been widely observed in neural assemblies in cortical areas during working memory or decision making [19,21,22]. In the literature, RNNs have been used to generate neural sequences [4,23,24]. However, these sequences were trained using supervised learning; it remains unknown whether rule-specific neural representations or sequences can emerge from a trained RNN. It is also unclear how these neural representations change due to network excitation and inhibition. Inspired by these questions, we developed an excitatory-inhibitory SRNN model that satisfies Dale’s principle [7,25], and we adapted both a SuperSpike surrogate gradient learning algorithm [17] and spike frequency adaptation (SFA) method [26,27] to train the SRNN. Additionally, unlike the rate-based RNN, SRNN can provide millisecond resolution to characterize neuronal tuning.

The contributions of this paper are threefold. First, to alleviate the vanishing gradient problem, we adapted the SuperSpike surrogate gradient algorithm to learn excitatory-inhibitory SRNN parameters. This combined computational strategy is key to efficiently training the SRNN for delayed working memory tasks and successfully replicating experimental findings. Second, we showed the trained SRNN produced emergent task-relevant, neural representations of excitatory and inhibitory neurons, such as neural sequences and oscillatory spiking activity during the delay period. Our computer model’s predictions match the published experimental data. Third, we manipulated and quantified the impact, of excitatory-to-inhibitory (E/I) balance, delay duration, and cortical connectivity on both task performance and neural representations. Overall, our large-scale SRNN modeling and simulations provide a computational framework to study task representations and dynamics of prefrontal circuits in a wide range of cognitive tasks.

## METHODS

### Animal Behaviors and Electrophysiological Recordings

**Figure 1:**
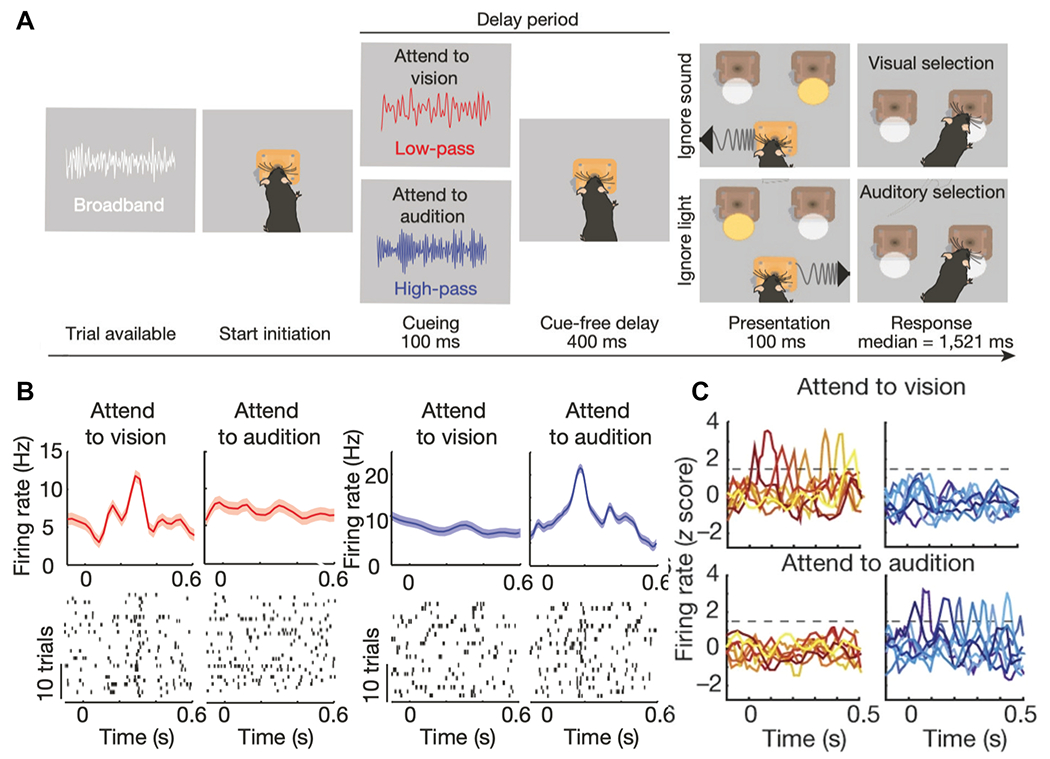
Rule-specific sequential PFC activity emerges during a mouse working memory task. (A) Schematic of 2AFG task. (B) Representative peri-stimulus time histogram (PSTH) and spike rasters for mouse PFC neurons with specific tuning of attending to vision (rule 1) or attending to audition (rule 2). Time 0 denotes the trial onset or the start of cue. (C) Examples of tuning peaks during 0-500 ms from mouse PFG neurons across multiple sessions (Figures reproduced from Schmitt et al., 2017, with permission, ©Springer Nature).

In the 2AFC task, mice were trained to initiate a trial upon receiving broadband white noise [19]. Upon successful initiation, white noise was immediately replaced by either low-pass or high-pass filtered noise for 100 ms to indicate the rule (i.e., attending to vision vs. audition). This was followed by a delay period lasting ~ 400 ms before the target stimuli presentation. The animals in the experiment would then make a decision (visual vs. auditory selection) by choosing one of two response ports following a 100-ms stimulation presentation after the delay period. Animals received a reward if their behaviors led to a correct rule-specific response at two specific response ports (Fig. 1A). In an extension of the 2AFC task, animals were trained to respond to four response ports instead of two response ports (known as 4AFC task), whereas the other task phases remained unchanged. Responses were scored as correct or one of three different error types (executive error, sensory error, or both).

Details of experimental protocols, animal behaviors and electrophysiological recordings have been published [19]. All animal experiments were performed according to the guidelines of the US National Institutes of Health and the Institutional Animal Care and Use Committee (IACUC) at the New York University Langone Medical Center.

### Computer Simulation Paradigm Based on SRNN

**Figure 2:**
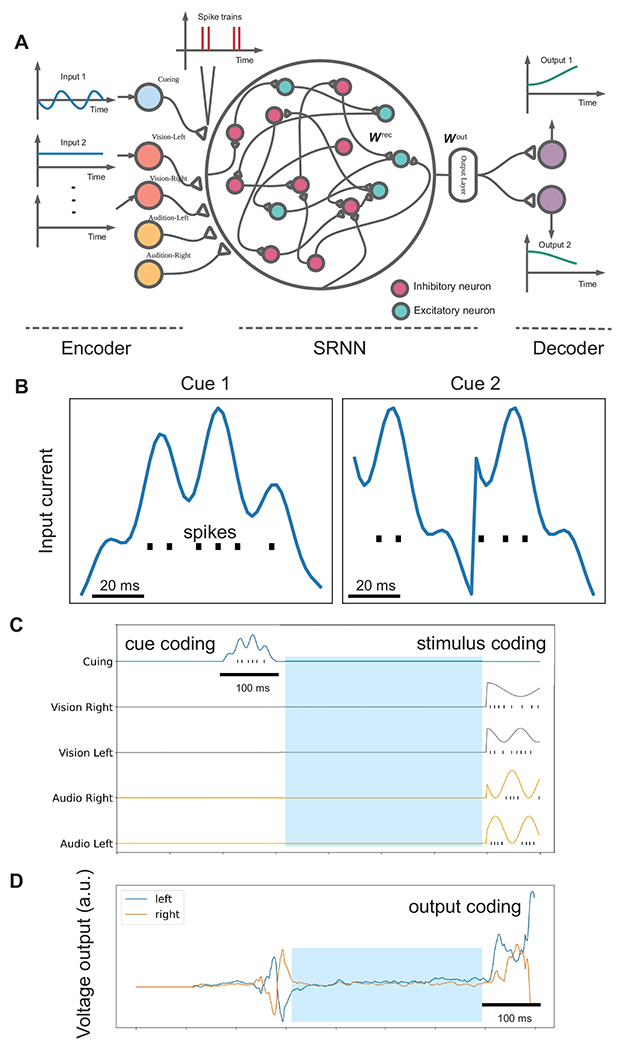
Schematic of computer simulations: an input encoder, a SRNN and an output decoder. (A) The SRNN consisting of both excitatory and inhibitory spiking neurons, receives the input spike trains converted from an encoder, and generates the output voltage and spike trains that are fed into a decoder, which generates the final decision. The neurons are fully connected with recurrent weights ***W***^rec^, and the readout layer was parameterized by ***W***^out^. (B) Illustration of encoding two cueing current inputs into spikes. (C) Illustration of cue encoding (cue 1) and four different sensory inputs. Shade areas denotes the 400-ms delay period in between cueing and stimulus presentation. (D) The decoder integrates and transforms the spike trains from the SRNN into an analog signal. The final voltage values of two readout neurons are shown.

To mimic the rule-dependent computation in the PFC circuit, we set up a computational model to perform the described cognitive task (Fig. 1). The complete model consisted of three independent components: an input encoder, the SRNN and an output decoder, as shown in Fig. 2A. The role of the input encoder was to transform the analog input signal into spikes. We used five leaky LIF neurons for representing the 2AFC task: one neuron for generating cueing or rule inputs, and the other four neurons for encoding distinct sensory cues (e.g., Vision/Left, Vision/Right, Audition/Left, Audition/Right as in [19], Fig. 2B). Two rules in this task were attending to vision (Rule 1) versus attending to audition (rule 2), where the context cues were represented by two different cues (cue 1 versus cue 2). The role of the output decoder was to integrate and transform the spikes from the SRNN into an analog signal for the final (binary) decision. We used two LIF neurons for the 2AFC task. The final voltage values of readout neurons were interpreted as the probabilities of a binary decision. An illustration of input-output encoding is shown in Fig. 2C,D.

To mimic the behavioral task in rodent experiments, we used the following computer simulation setup:

- 200-ms fixation period, during which only white noise was supplied.
- 100-ms cueing period, during which the spike trains were generated by the cueing encoder and sent to the SRNN.
- 400-ms cueing delay period, during which only white noise was supplied.
- 100-ms stimulus presentation period, during which two spike trains were represented: one from the visual channel, and the other from the auditory channel.
- Response period, during which the cost function was computed at the end of the presentation period, and the network back-propagated the error and computed the gradient.

We simulated independent Monte Carlo trials based on different initial conditions for each rule condition. In what follows, we present the mathematical details and implementation of each component. The mathematical symbols and notations are summarized in Table 1.

### Mathematical description of the SRNN

The structure of an SRNN is similar to a rate-based RNN [7], except that each unit of the SRNN consists of a biologically-constrained LIF neuron. All neurons were connected through a recurrent weight matrix ***W***^rec^, and the SRNN received cueing/sensory inputs from the encoders via ***W***^cueing^ and ***W***^sensory^, and produced outputs through the output weight matrix. The basic dynamics of neurons in SRNN was described as follows:

(1a) $$\frac{dV_{i}^{\mathrm{rec}}}{dt}=\frac{1}{\tau_{\text{m}}}\left(\left(v_{\text{rest}}-V_{i}^{\text{rec}}(t)\right)+I_{i}^{\text{rec}}(t)R\right)+\xi_{i}^{\text{m}}$$

(1b) $$\frac{dI_{i}^{\text{rec}}}{dt}=-\frac{I_{i}^{\text{rec}}(t)}{\tau_{\text{syn}}}+\overset{N_{\mathrm{cue}}}{\underset{c=1}{\sum }}W_{ic}^{\text{cueing}}S_{c}^{\text{cueing}}(t)+\overset{N_{\text{sensory}}}{\underset{k=1}{\sum }}W_{ik}^{\text{sensory}}S_{k}^{\text{sensory}}(t)+\overset{N_{\text{rec}}}{\underset{j=1}{\sum }}W_{ij}^{\text{rec}}S_{j}^{\text{rec}}(t)+I_{i}^{\text{base}}(t)$$

(1c) $$S_{i}^{\text{rec}}(t)=\text{Θ}\left(V_{i}^{\text{rec}}(t)-V_{i}^{\text{th}}(t)\right)$$

where *R* denotes the resistance, *V* denotes the voltage, and *I* denotes the current. Each neuron was modeled by a leaky integrator, where Θ denotes the Heaviside step function. In Eq. 1a, the dynamics of membrane potential $V_{i}^{\text{rec}}$ is driven by synaptic current *I_i_* and zero-mean white noise $\xi_{i}^{m}$ and compared with its resting state *v*_rest_. In Eq. 1b, the synaptic current current *I_i_* was modeled by a linear sum of all presynaptic spikes through three associated weight matrices $\left\{W_{ic}^{\text{cueing}},W_{ik}^{\mathrm{sensory}},W_{ij}^{\text{rec}}\right\}$. Spike trains $S_{c}^{\text{cueing}}$ and $S_{k}^{\text{sensory}}$ originated from the *c*-th cueing encoder and the *k*-the sensory encoder, respectively; and *S*^rec^ represented the spike trains from other recurrently connected neurons. The current $I_{i}^{\text{base}}$ was injected to maintain a baseline firing rate. Finally, a spike $S_{i}^{\text{rec}}$ would fire when *V_i_* was above the spike threshold $V_{i}^{\text{th}}$. After spiking, $V_{i}^{\text{rec}}$ was reset to membrane potential resting state *v*_rest_ with a short refractory period *τ*_ref_.

We added additional random noise to the SRNN to emulate the noisy nervous system. Two sources of noise were considered such that the SRNN still maintained a reasonable level of neuronal firing rate even in the absence of external inputs. The first one was the voltage noise *ξ*^*m*^ (Eq. 1a), which was sampled from a zero-mean normal distribution. The second type of noise was the baseline current noise $\xi_{i}^{\text{base}}$ (Eq. 2), which was also sampled from a zero-mean normal distribution at every step by following a random-walk model for the baseline current $I_{i}^{\text{base}}$:


(2) $$I_{i}^{\text{base}}(t+dt)=I_{i}^{\text{base}}(t)+\xi_{i}^{\text{base}}$$

Our SRNN has several important features: (i) the SRNN consisted of excitatory and inhibitory neurons that follow Dale’s principle; (ii) the SRNN implemented an efficient SuperSpike algorithm [17] to alleviate the vanishing gradient problem; (iii) the SRNN employed SFA to strengthen the memory capacity during the delay period [26]; and (iv) the SRNN used a regularized cost function that penalized the excessive spikes in the neuronal population (thereby imposing a sparsity constraint).

### Network initialization

We initialized the neuronal states and neuronal baseline firing rates using the following procedure. First, we uniformly randomized the initial membrane potential for every neurons within a reasonable range (between the membrane potential resting state *v*_rest_ and the baseline spiking threshold *v*_th_) at each trial. This range was chosen to improve the robustness of the model. Second, the initial baseline current for each neuron was sampled from a truncated normal distribution, which did not vary across different trials (Table 2).

All the connection weights of the SRNN were firstly initialized via the Glorot method [28]. Given a matrix with the size of fan_in_ × fan_out_, every connection weight *w*_init_ was sampled from a uniform distribution as follows

(3) $$w_{\text{init}}∼U\left(-\sqrt{\frac{6}{{\text{fan}}_{\text{in}}+{\text{fan}}_{\text{out}}}},\sqrt{\frac{6}{{\text{fan}}_{\text{in}}+{\text{fan}}_{\text{out}}}}\right)$$

### Encoder and decoder

Both the encoder and decoder (Fig. 2A) followed the same LIF neuron structure; however, in contrast to the SRNN, we did not impose neither Dale’s principle nor SFA on the encoder and decoder. Specifically, we assumed the same dynamics and hyperparameters setup for the membrane potential and spike generation.

(4a) $$\frac{dV_{i}^{\text{encoder}}}{dt}=\frac{1}{\tau_{\text{m}}^{\text{encoder}}}\left(\left(v_{\text{rest}}^{\text{encoder}}-V_{i}^{\text{encoder}}(t)\right)+I_{i}^{\text{encoder}}(t)R^{\text{encoder}}\right)$$

(4b) $$S_{i}^{\text{encoder}}(t)=\text{Θ}\left(V_{i}^{\text{encoder}}(t)-v_{\text{th}}^{\text{encoder}}(t)\right)$$

(4c) $$\frac{dV_{i}^{\text{decoder}}}{dt}=\frac{1}{\tau_{\text{m}}^{\text{decoder}}}\left(\left(v_{\text{rest}}^{\text{decoder}}-V_{i}^{\text{decoder}}(t)\right)+I_{i}^{\text{decoder}}(t)R^{\text{decoder}}\right)$$

(4d) $$S_{i}^{\text{decoder}}(t)=\text{Θ}\left(V_{i}^{\text{decoder}}(t)-v_{\text{th}}^{\text{decoder}}(t)\right)$$

(4e) $$\frac{dI_{i}^{\text{decoder}}}{dt}=-\frac{I_{i}^{\text{decoder}}(t)}{\tau_{\text{syn}}^{\text{decoder}}}+\sum W_{io}^{\text{out}}S_{o}^{\text{rec}}(t)$$

where $S_{o}^{\text{rec}}$ represents the spiking output from the excitatory neuronal population in the SRNN. $I_{i}^{\text{encoder}}$ was provided by the continuous-valued cueing or sensory input (Fig. 2B), whereas the dynamics of $I_{i}^{\text{decoder}}$ was driven by the output of SRNN weighted by an output matrix ***W***^out^ (Eq. 4e). Finally, the decoder output produced a two-dimensional continuous-valued voltage vector **V**^decoder^, which represented the probability of making corresponding decisions (e.g., [0, 1] representing the left choice and [1, 0] representing the right choice). The default parameters of the encoder and decoder are listed in Table 3. Here we assumed the same parameter setup between the encoder and the decoder. However, the task performance were not sensitive to the exact choice of these parameters, and they can be easily modified based on the specific form of cue input or spiking frequency.

### Imposing biological constraints

#### Dale’s principle

Similar to the previous work [7], we imposed Dale’s principle onto the SRNN. Specifically, cortical neurons have either purely excitatory or inhibitory effects on postsynaptic neurons, and the ratio *ϕ*_exc_ of excitatory cortical neurons to all the neurons was 80%.

According to the excitatory (E) and inhibitory (I) populations, the recurrent weight matrix **W**^rec^ was decomposed into four blocks: {***W***_EE_, ***W***_EI_, ***W***_IE_, **W**_II_}. The elements in ***W***_EE_ and ***W***_IE_ were all positive, representing the excitatory-to-excitatory and excitatory-to-inhibitory connections, respectively; whereas the elements in ***W***_II_ and ***W***_EI_ were all negative, representing the inhibitory-to-inhibitory and inhibitory-to-excitatory connections, respectively. We initialized $W_{\mathrm{rand}}^{\mathrm{rec}}$ through the Glorot method (Eq. 3), and then rescaled it based on its eigenvalue spectra [1, 29]. Specifically, let $\rho =\max\left\{\left|\lambda_{1}\right|,\ldots ,\left|\lambda_{n}\right|\right\}$ denote the largest absolute value of eigenvalues of $W_{\text{rand}}^{\text{rec}}$; we scaled $W_{\text{rand}}^{\text{rec}}$ into $W_{\text{init}}^{\text{rec}}$ by *g/ρ* such that as

(5) $$W_{\text{init}}^{\text{rec}}=g/\rho \cdot W_{\text{rand}}^{\text{rec}}$$

where *g* = 1.5.

We further generated a mask matrix ***D*** = {*D_ij_*} to impose Dale’s principle on $W_{\text{init}}^{\text{rec}}$ such that $W^{\text{rec}}=\left|W_{\text{init}}^{\text{rec}}\right|⊙D$, where Θ denotes the element-wise product. Specifically, we set *ϕ*_exc_ = 0.8 to reflect the 4:1 ratio of excitatory-to-inhibitory neurons, and assumed that each neuron has its own constraint item

(6) $$D_{ij}=\{\begin{array}{ll} \frac{d^{\mathrm{exc}}}{d^{\mathrm{norm}}}, & j\leq \phi_{\mathrm{exc}}N_{\mathrm{rec}}\cap i\neq j\\ \frac{d^{\mathrm{ihn}}}{d^{\mathrm{norm}}}, & j>\phi_{\mathrm{exc}}N_{\mathrm{rec}}\cap i\neq j\\ 0, & i=j \end{array}$$

where *d*^exc^ and *d*^inh^ denote the constraints for E and I neurons, respectively; namely, *d*^exc^ was used for ***W***_EE_ and ***W***_IE_, and *d*^inh^ was used for ***W***_II_ and ***W***_EI_; there was no self-connection so that *D*_ii_ = 0. Similar to [7,30], we set *d*^exc^ = 1 and $d^{\text{inh}}=-\frac{\phi_{\text{exc}}}{1-\phi_{\text{exc}}}$ in order to keep the balance of excitation and inhibition (i.e., $\sum_{\text{exc}}d^{\mathrm{exc}}+\sum_{\text{inh}}d^{\text{inh}}=0)$). Furthermore, *d*^norm^ is a normalizing constant: $d^{\text{norm}}=\sqrt{\sum_{\text{exc}}{\left(d^{\text{exc}}\right)}^{2}+\sum_{\text{inh}}{\left(d^{\text{inh}}\right)}^{2}}$.

#### Spike frequency adaptation (SFA)

SFA is a dynamic self-inhibition mechanism that allows neurons to adapt their spiking threshold to decrease firing. Recently, it has been shown that SRNNs with SFA can significantly increase the longer short-term memory capability [26]. Briefly, let *A*_i_ denote an adaptation term for the spiking threshold. The dynamics of *A_i_*(*t*) was described as follows

(7a) $$A_{i}(t)=\varphi A_{i}(t-dt)+(1-\varphi )S_{i}(t-dt)$$

(7b) $$V_{i}^{\text{th}}(t)=\psi A_{i}(t-dt)A_{i}^{\text{mask}}+v_{\text{th}}$$

where *ψ* > 0 is a scale parameter. Specifically, when the *i*-th neuron bred, *A_i_* increased instantly; otherwise, it decayed with a factor of $\varphi =\exp\left(-\frac{dt}{\tau_{a}}\right)$ (Eq. 7a), where *τ*_a_ = 400 ms denotes the adaptation time constant (*τ*_a_ ≫ *τ*_m_ and *τ*_a_ ≫ *τ*_syn_). Furthermore, the voltage spiking threshold $V_{i}^{\text{th}}(t)$ was adjusted based on *A_i_*(*t*) and a binary mask $A_{i}^{\text{mask}}$. From a total of *N*_rec_ neurons, we randomly selected 25% of population and assigned them with SFA ($A_{i}^{\text{mask}}=1$). As discussed in [26], neurons with SFA introduce a spread of longer time constants into the SRNN and increase the long short-term memory (LSTM) capacity.

#### SRNN Training

The decoder output $V^{\text{decoder}}=\left\{V_{1}^{\text{decoder}},V_{2}^{\text{decoder}}\right\}$ was a two-dimensional continuous-valued vector representing the voltage values from the decoder’s neurons. Let ***y*** = {*y*_1_, *y*_2_} denote the corresponding target vector output; we used the mean-squared error as the cost function L, which summed over all single trials loss $L_{\ell }$ using a batch size *N*_batch_:

(8) $$L_{\text{mse}}=\frac{1}{N_{\text{batch}}}\overset{N_{\text{batch}}}{\underset{\ell =1}{\sum }}L_{\ell }=\frac{1}{2\times 5\times N_{\text{batch}}}\overset{N_{\text{batch}}}{\underset{\ell =1}{\sum }}\overset{2}{\underset{i=1}{\sum }}\overset{N_{\text{step}}}{\underset{t=N_{\text{step}}-4}{\sum }}{\left(y_{i,l}(t)-V_{i,l}^{\text{decoder}}(t)\right)}^{2}$$

where *ℓ* is the trial index, and *N*_step_ = *T/dt* is the total number of time steps needed within a single trial. We used the last 5 time steps during the sensory presentation period to compute the error.

In addition, we incorporated a firing rate regularization term $L_{\mathrm{fr}}$ into the cost function, which penalized the mean firing rate of neuronal population. This regularization avoided the excessive firing frequency, also prevented the bring rate of excitatory neurons higher than that of inhibitory neurons. Specifically, the regularized cost function was written as

(9a) $$L=L_{\text{mse}}+\lambda L_{\text{fr}}$$

(9b) $$L_{\text{fr}}=\overset{N_{\mathrm{bin}}}{\underset{b=1}{\sum }}{\left(\frac{1}{N_{\text{rec}}N_{\text{batch}}}\overset{N_{\mathrm{rec}}}{\underset{j=1}{\sum }}\overset{N_{\text{batch}}}{\underset{\ell =1}{\sum }}r_{j,\ell ,b}\right)}^{2}$$

where *r_j,ℓ,b_* denotes the instantaneous firing rate of the *j*-th neuron at the *b*-th temporal bin (1 bin = 5 × *dt* = 10 ms and *N*_bin_ = *N*_step_/5 = 80) in the *ℓ*-th trial; and λ denotes the regularization coefficient.

We further used the gradient descent algorithm (such as Adam [31]) to train all weight matrices of SRNN:

(10) $$W_{ij}^{\left(n\right)}=W_{ij}^{\left(n-1\right)}-\eta {\left(\frac{\partial L}{\partial W_{ij}}\right)}^{\left(n-1\right)}$$

where *η* > 0 is a learning rate parameter, and ${\left(\frac{\partial L}{\partial W_{ij}}\right)}^{\left(n-1\right)}$ denotes the gradient evaluated on the parameter *w_ij_* at iteration *n* – 1. In BPTT, the computation of gradient back-propagation was defined by the chain rule:

(11) $$\frac{\partial L}{\partial W^{\text{rec}}}=\frac{\partial L}{\partial V^{\text{decoder}}}\frac{\partial V^{\text{decoder}}}{\partial I^{\text{decoder}}}\frac{\partial I^{\text{decoder}}}{\partial S^{\text{rec}}}\frac{\partial S^{\text{rec}}}{\partial W^{\text{rec}}}\;=\frac{\partial L}{\partial V^{\text{decoder}}}\frac{\partial V^{\text{decoder}}}{\partial I^{\text{decoder}}}\frac{\partial I^{\text{decoder}}}{\partial S^{\text{rec}}}\frac{\partial S^{\text{rec}}}{\partial V^{\text{rec}}}\frac{\partial V^{\text{rec}}}{\partial I^{\text{rec}}}\frac{\partial I^{\text{rec}}}{\partial W^{\text{rec}}}$$

where $\frac{\partial S^{\text{rec}}}{\partial W^{\text{rec}}}=\frac{\partial S^{\text{rec}}}{\partial V^{\text{rec}}}\frac{\partial V^{\text{rec}}}{\partial I^{\text{rec}}}\frac{\partial I^{\text{rec}}}{\partial W^{\text{rec}}}$ denotes the back-propagated gradient within the SRNN. Therefore, the dynamics of the *i*-th neuron was described by the following equations

(12a) $$S_{i}(t)=\text{Θ}\left(V_{i}(t)-V_{\text{th}}\right)$$

(12b) $$V_{i}(t)=\exp\left(\frac{-dt}{\tau_{\text{mem}}}\right)\left(V_{i}(t-dt)\right)+V_{\text{rest}}+I_{i}(t-dt)R$$

(12c) $$I_{i}(t)=\exp\left(\frac{-dt}{\tau_{\text{syn}}}\right)I_{i}(t-dt)+W_{ij}S_{j}(t-dt)$$

In the chain rule, the corresponding three derivatives were given by

(13a) $$\frac{\partial S_{i}(t)}{\partial W_{ij}}=\frac{\partial \text{Θ}\left(V_{i}(t)-V_{\text{th}}\right)}{\partial V_{i}(t)}\frac{\partial V_{i}(t)}{\partial W_{ij}}$$

(13b) $$\frac{\partial V_{i}(t)}{\partial W_{ij}}=\exp\left(\frac{-dt}{\tau_{\text{mem}}}\right)\frac{\partial V_{k}(t-dt)}{\partial W_{ij}}+\frac{\partial I_{i}(t-dt)R}{\partial W_{ij}}$$

(13c) $$\frac{\partial I_{i}(t)}{\partial W_{ij}}=\exp\left(\frac{-dt}{\tau_{\text{syn}}}\right)\frac{\partial I_{i}(t-dt)}{\partial W_{ij}}+S_{j}(t-dt)$$

Notably, because of using the Heaviside step function Θ, the gradient information $\frac{\partial \text{Θ}}{\partial V_{i}}$ was zero in the absence of spiking [18]. Therefore, the gradient $\frac{\partial L}{\partial W^{\text{rec}}}$ propagated from its previous layers or time steps suffered from the vanishing gradient. To prevent such gradient vanishment, we replaced the gradient in the chain rule by a surrogate gradient known as SuperSpike surrogate gradient [17], which is described below.

#### SuperSpike Surrogate Gradient

The SuperSpike is a nonlinear voltage-based three-factor learning rule, which is capable of training multilayer networks of deterministic LIF neurons to perform nonlinear computations on spatiotemporal spike patterns [17]. Specifically, we computed the SuperSpike surrogate gradient as follows

(14) $$\frac{\partial S_{i}(t)}{\partial W_{ij}}\approx \frac{\partial \sigma \left(V_{i}(t)-V_{\text{th}}\right)}{\partial V_{i}(t)}\frac{\partial V_{i}(t)}{\partial W_{ij}}\;=\frac{1}{{\left(1+\beta \left|V_{i}(t)-V_{\text{th}}\right|\right)}^{2}}\frac{\partial V_{i}(t)}{\partial W_{ij}}$$

where $\sigma (x)=\frac{x}{1+|x|}$ denotes the so-called fast sigmoid function; *β* is a scaling parameter that defines the sharpness of the approximate derivative: the greater *β* is, the sharper the derivative becomes. In Eq. 14, we approximated $\frac{\partial \text{Θ}\left(V_{i}(t)-V_{\text{th}}\right)}{\partial V_{i}(t)}$ with a smooth surrogate gradient $\frac{\partial \sigma \left(V_{i}(t)-V_{\text{th}}\right)}{\partial V_{i}(t)}$.

The computation of SuperSpike gradient has been interpreted as Hebbian coincidence detection [17]. Specifically, the gradient computation of $\frac{\partial S_{i}(t)}{\partial W_{ij}}$ consists of the product of two derivative terms (Eq. 14), the first-order derivative term $\frac{\partial \sigma \left(V_{i}(t)-V_{\text{th}}\right)}{\partial V_{i}(t)}$ corresponds to the dynamics of the postsynaptic neuron of *W_ij_*. The second partial derivative term $\frac{\partial V_{i}(t)}{\partial W_{ij}}$ approximates the concentration of neurotransmitters at the synapse *W_ij_*, which represents the presynaptic neuron activity. Thus, training based on SuperSpike gradient is considered as an analogue of Hebbian learning.

### Identification of Rule-Specific Neuronal Tuning

Given single-trial spike train simulations of each neuron, we displayed the spike rasters and computed the peri-stimulus time histogram (PSTH) using a 8-ms bin size (i.e., 4 bins). To obtain the smooth tuning profile, the PSTH was convolved with a Gaussian kernel (with kernel width of 4) to create a spike density function based on 50 simulated trials, which was further Z-scored by subtracting the mean firing rate during the delay period (for both rules) and dividing the standard deviation (SD) over the same period. The neurons with Z-scored peak firing rate above a threshold were considered to have sharp tuning. We first screened the neurons with one or more sharp candidate peaks during the delay period for the subsequent analysis. Neurons with very low mean firing rates (< 2 Hz) during the delay period were not considered in the tuning analysis. We used a similar criterion as [19] to identify the genuine tuning peaks. In addition to the minimum firing rate criterion, these candidate peaks needed to have Z-score values greater than a threshold (e.g., 2.33 for *p* < 0.01). We empirically used a threshold of 2 to match the experimental data. We further categorized the neurons with putative tuning peaks depending on the number of peaks. The majority of identified neurons with rule-specific tuning had 1-2 peaks.

To characterize the maximum peak compared to the baseline noise, we computed the peak-to-noise ratio (PNR) for each simulated unit, and compared them between two rules. We defined the peak as the maximum Z-scored firing rate during the delay period and the noise as the standard deviation of Z-scored firing rate excluding the peak region. For noise estimation, we used the code https://pyastronomy.readthedocs.io/en/latest/pyaslDoc/aslDoc/estimateSNR.html. A large PNR value indicates either a high peak firing rate or a low noise variance.

### Characterization of Sequential Activation

We computed the sequentiality index (SI) to characterize the sequential activation of the population response of excitatory neurons during the delay period. For a given trial, the SI was defined as the sum of the entropy of the peak response time distribution {*P_b_*(*t*_peak_)} (where $\sum_{b}P_{b}\left(t_{\text{peak}}\right)=1$) of the recurrent neurons and the mean log ridge-to-background ratio of the neurons, where the ridge-to-background ratio for a given neuron was defined as the mean activity of the neuron inside a small window around its peak response time FR*_i_* (inside peak window) divided by its mean activity outside this window FR*_i_* (outside peak window) [22,32]:

$$\text{SI}=-\underset{b}{\sum }P_{b}\left(t_{\text{peak}}\right)\log P_{b}\left(t_{\text{peak}}\right)+\underset{i}{\sum }\log\frac{{\text{FR}}_{i}(\text{inside peak window})}{{\text{FR}}_{i}(\text{outside peak window})}$$

The computer code for computing the SI was available online (https://github.com/eminorhan/recurrent-memory/blob/087a4a67b684ca2e8b6d8102c4221c88105cc16f/utils.py#:L16). To test the statistical significance of median SI differences between two conditions, we used a nonparametric rank-sum test throughout the study.

### Dimensionality Reduction

To investigate the neuronal population response, we used the principal component analysis (PGA) to extract the principal components of population responses during the delay period. To compute the neural representation or trajectories in the state space, we projected the data, onto the subspaces of dominant principal components (PCs). and obtained the low-dimensional latent state trajectory **x**(*t*). Furthermore, we defined the network kinetic energy to characterize the dynamics of SRNN [33]

(15) $$K(\text{x})=\frac{1}{2}\|\overset{\cdot }{x}(t){\|}^{2}$$

A low value of K(x) indicates the state reaches a slow-point or fixed-point region with low or zero momentum.

### Population Decoding

We used a LSTM-RNN model to read out the neuronal population responses during the delay period. The goal of the binary LSTM classifier is to predict a category of the temporal sequence output. We used two LSTM layers for binary classification and trained the LSTM with BPTT. To avoid overfitting, we used dropout (probability of 0.2) and *L*_2_ regularization (regularization parameter of 0.001). However, the performance of LSTM was insensitive to the choice of hyperparameters.

Upon completing training, we produced a total of 200 trials (evenly for each rule/stimulus configuration) for classification analysis. We temporally binned the spike trains (20 or 50 ms bin size) during the delay period. To train the LSTM classifier, we used the PCs as the features. The input to the LSTM was a vectorized input consisting of time bins-by-PCs features. The purpose of PCA was to reduce the number of features and avoid overfitting. We randomly split 600 trials into two groups, 90% used for training and 10% used for testing. We conducted 10-fold cross-validation and then computed the average test accuracy. The default classification threshold was 0.5.

### Computer Simulation of Local Field Potential (LFP)

Following the LFP simulation strategies [34,35], we generated a “LFP proxy” from the trained SRNN model. From *N* = 500 neurons, we computed the linear sum of synaptic currents of LIF neurons. For visualization, we plotted the single-trial time trace and computed the spectrogram of simulated LFP.

### Software Implementation

The Euler method was used in numerical simulations for integrating the differential equations. We used *dt* = 2 ms and scaled the noise appropriately. The SRNN implementation was built upon the Python package Norse (https://github.com/norse/norse), which is a deep learning library for spiking neural networks. Norse expands PyTorch with primitives for biologically inspired neural components, bringing two advantages: a modern and proven infrastructure based on PyTorch and deep learning-compatible spiking neural network components. We also adapted and updated the Python code (https://github.com/xjwanglab/pycog) on the weight update with the Dale’s rule constraint. Our custom software implementation can be found on GitHub (https://github.com/Jakexxh/srnn-2afc). To help the reader to repeat the experiments, we also provided a pre-trained checkpoint file.

## RESULTS

### Training SRNN for the 2AFC Task

We trained a biologically-inspired excitatory-inhibitory SRNN to perform the 2AFC task (Fig. 2). In the default setup (*N*_rec_ = 500), we used 400 excitatory neurons and 100 inhibitory neurons in the SRNN. We trained the SRNN on the Oracle’s cloud-based GPU-accelerated virtual machine (NVIDIA GPU P100, 16 GB GPU memory, 12 CPU cores, 78 GB CPU memory) within 30-60 epochs (200 batches per epoch), the performance gradually reached 100% (Fig. S1A,B). Based on different random initial conditions, we repeated the training procedure 10 times and obtained 10 independent realizations of trained SRNN. Each trained network was treated as one set of independent data. In total, we examined the task representations of 500 × 10 = 5000 simulated (4000 excitatory and 1000 inhibitory) neurons, and used them in the subsequent analyses. The initial distribution of synaptic weights (absolute value) was close to a uniform distribution. After learning, the weight distribution had a long-tailed behavior, and many of synaptic strengths were close to zeros (Fig. S1C).

Spike frequency adaptation (SFA) applies the situation where in response to a stimulus of constant intensity, many neurons initially respond with a high spike frequency and then decay down to a lower steady-state bring rate. Compared to the training algorithm with SFA, the SRNN trained without SFA typically did not converge within 100 epochs, resulting a poor classification accuracy of 75% (i.e., learning one rule, while guessing the other rule with 0.5 probability).

### Rule-Specific Neuronal Representations

**Figure 3:**
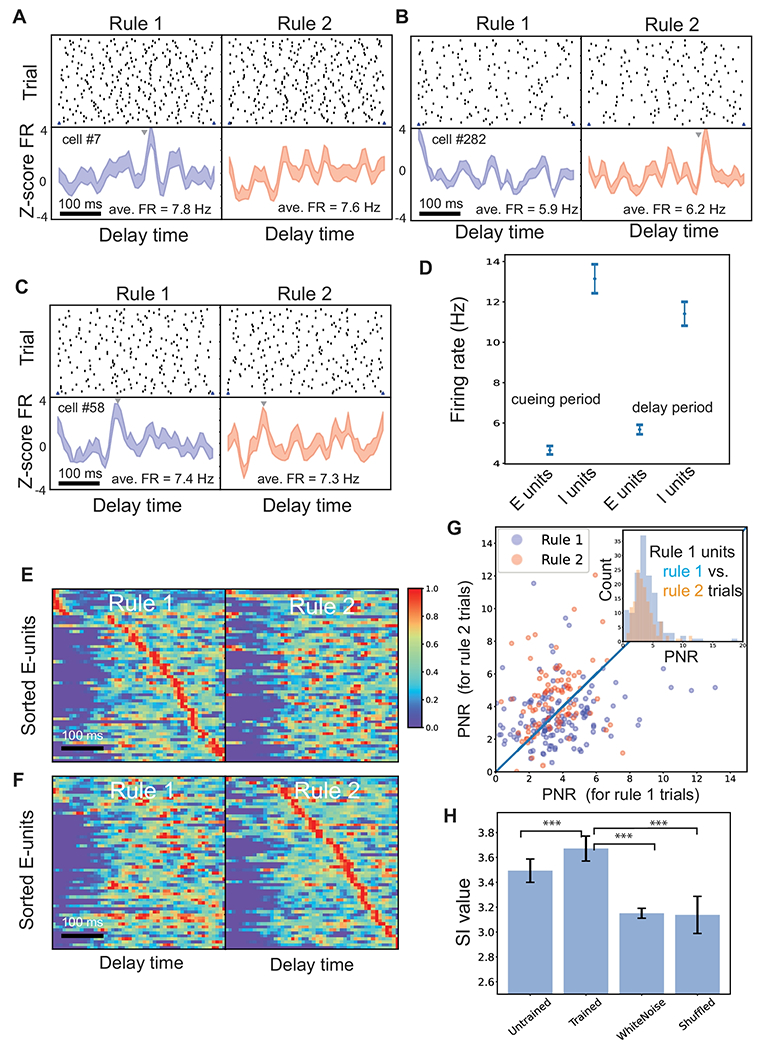
Rule-specific neural representations in the trained SRNNs. (A-C) Representative spike rasters and PSTHs of two task-modulated neurons for rule representation. Arrows indicate the peak firing for rule tuning. Panel A shows a representative neuron that has sharp tuning to rule 1, but not rule 2; panel B shows a representative neuron that has sharp tuning to rule 2, but not rule 1; panel C shows a representative neuron that has sharp tunings (but at different timings) with respect to two rules. Shaded area denotes SEM. In addition to the Z-score firing rate (FR), the mean FR during delay period is marked in the PSTH panel. (D) Statistics of averaged population firing rates during baseline (fixation) and delay periods. Error bar shows SEM across all neurons. (E,F) Heat maps (each row was normalized between 0 and 1) of normalized task-modulated excitatory neuronal firing rates from one trained SRNN. Note that the sorted E-neurons formed a “neural sequence” for one rule, but not the other. In this example, the sequentiality index (SI) was 3.78. (G) Comparison of the peak-to-noise ratio (PNR) of computer-simulated units between two rules. (H) Comparison of SI statistics for excitatory neurons between different conditions. Mean±SD statistics were computed from 10 untrained and 10 trained SRNNs. ***, *p* < 10^−3^.

After the SRNN learned the task, we first examined the neuronal responses at the single-unit level. We identified task-modulated excitatory neurons that showed sharp peaks during the delay period. We found some excitatory neurons showed rule-specific tunings, with typical differential peaks or peak locations between two rules (Fig. 3A,B). As seen in the spike raster examples, some neurons showed sharp tuning at specific timing for coding one rule, but not the other; but their average firing rates during the task delay were similar. At the population level, the number of peaks was comparable between two rules. A smaller percentage of units were tuned to both rules, with distinct temporal offset in tuning peak (Fig. 3C). This emergent property was reminiscent of the experimental finding in rodent PFC neuronal recordings (Fig. 1B). The ratio of task-modulated or rule-specific tuning excitatory neurons was averaged at 8.1% (over 10 realizations), qualitatively similar to experimental findings [19], and the ratio of excitatory neurons representing rule 1 and rule 2 was roughly equal (4.08% vs. 4.02%). In addition, the averaged firing rates of excitatory and inhibitory neurons during the delay period were 4.15 ± 0.21 Hz and 10.53 ± 0.56 Hz (mean±SEM), respectively. Interestingly, we observed a slight increase in the firing rate of excitatory neurons during the delay period as compared to the cue period, yet a dramatic firing rate decrease in inhibitory neurons (Fig. 3D).

Furthermore, we sorted the rule-specific excitatory neurons according to the peak timing at each rule (Fig. 3E,F). Together, their trial-averaged population responses (visualized as a normalized heat map) showed the emergence of sequential neural activity (comparing with Fig. 1C). The maintenance of working memory as an approximately sequential activation of rule-specific neurons during task delay is reminiscent of previous experimental findings [21,22]. Notably, neurons contributed to different roles in sequence representation at different rules. To quantify the variability of the sequential activation, we further computed the SI measure (see Methods) for rule-specific excitatory neurons as well as for the complete excitatory populations (Fig. S2). Furthermore, we also computed the maximum Z-scored peak-to-noise ratio (PNR; see Methods) of all simulated units and compared them between two rules. A high peak-to-noise ratio indirectly supported the evidence of rule tuning. In general, rule-1 tuned units had a greater PNR in rule-1 trials than in rule-2 trials, whereas rule-2 tuned units had a greater PNR in rule-2 trials than in rule-1 trials (Fig. 3G). As a control, we also compared the SI statistics between the trained and untrained networks based on the excitatory neuronal population (Fig. 3H). To obtain the chance-level statistic, we fed the white noise input to the trained SRNN and recomputed the SI based on excitatory neuronal activation during the delay period. The chance-level SI was significantly less than the task condition (*p* = 0.00015, rank-sum test). Additionally, we performed another control by randomly shuffling the trained recurrent weight matrix and repeated the SI analysis. Again, the shuffled SI statistic was significantly less than the task condition (*p* = 0.00016, rank-sum test).

**Figure 4:**
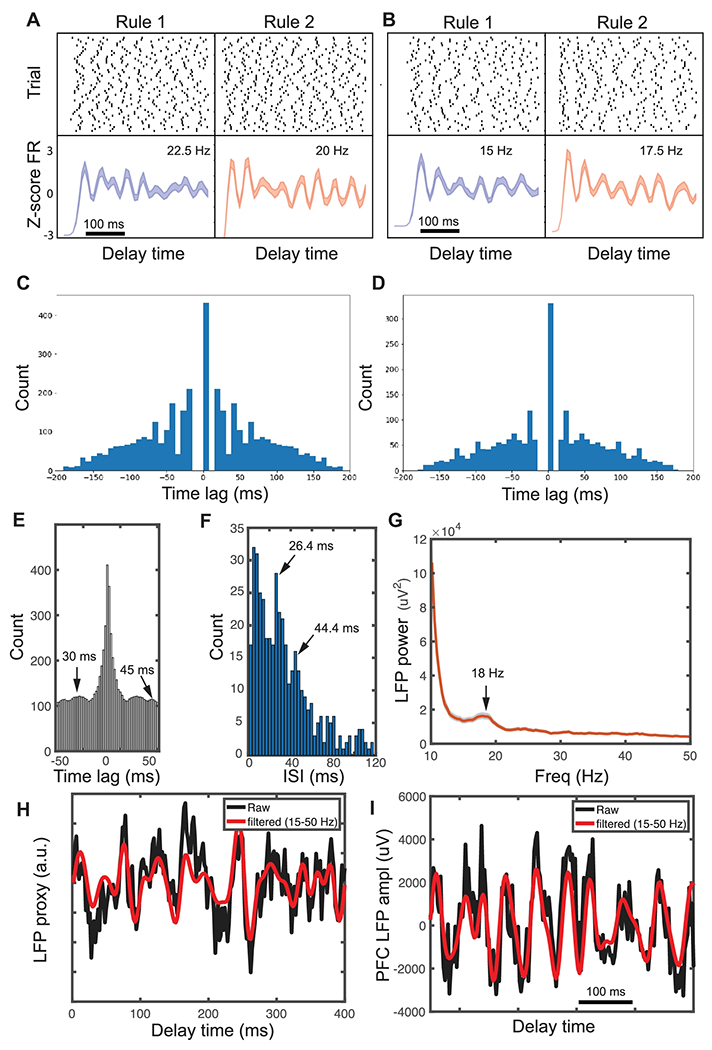
Spiking activities of inhibitory neurons from the trained SRNN showed beta oscillations. (A,B) Two examples of representative spike rasters and PSTHs that showed differential oscillatory frequency or phase in firing between two rules. Error bar represents SEM. The number shows the approximate oscillatory frequency. (C,D) Auto-correlograms of two neurons shown in panels A and B. (E) An Auto-correlogram example of mouse PFC neuron that showed rhythmic firing at the beta-low gamma range. (F) An inter-spike interval (ISI) histogram example of mouse PFC neuron that showed rhythmic firing. (G) Trial-averaged local field potential (LFP) spectrogram from the mouse PFC recording showed a beta oscillation during the task delay. Shaded area denotes SEM. (H) Single-trial LFP proxy was generated from the sum of synaptic currents of 500 neurons from the trained SRNN. A single-trial simulation of 400-ms trace during delay was shown. (I) A representative singe-trial LFP trace from the mouse PFC recording.

In addition, we observed rhythmic firing in the spiking activity of a subset of inhibitory neurons during the delay period. This was also independent of their task-tuned properties. A closer look at their PSTHs or auto-correlograms revealed strong beta (15-30 Hz) oscillations at the spiking activity (Fig. 4). In total, 25% (25/1000) of inhibitory neurons exhibited beta rhythms in their simulated spiking activities. Some inhibitory neurons also showed differential rule-specific firing, in terms of the oscillatory frequency or phase. However, we did not find any correlation between the power of this rhythmic activity and task performance. Fast-spiking (FS) interneurons are known to provide important feedback inhibition to generate fast (> 10 Hz) rhythmic oscillations through recurrent inhibition between excitatory and inhibitory populations [36]. Although beta rhythmic spiking was not emphasized in the original report [19], this might be a sampling issue. Nevertheless, some evidence of beta oscillatory activity could be found at both spiking (Fig. 4E,F and Fig. S3C) and local field potential (LFP, Fig. 4G) levels in the mouse PFC recordings. This phenomenon was also consistent with the report in task-performing monkeys during working memory tasks (see Discussion). Furthermore, we generated a LFP proxy based on the sum of synaptic currents of 500 neurons from the trained SRNN. As shown in the single-trial simulation of 400-ms trace during the delay period (Fig. 4H), the beta or low gamma oscillations were noticeable. This simulated LFP trace was qualitatively similar to the experimental PFC recording (Fig. 4I).

We have seen heterogeneous neuronal responses at the single-unit, level in both simulated and experimental data. Notably, we have shown the neural representations during 400-ms task delay period. Nevertheless, we could also visualize and compare the neural representations during 100-ms cueing and 100-ms stimulus presentation periods with experimental data (see examples in Fig. S3). However in the SRNN, the simulated spiking activity during cueing and stimulus presentation completely depended on the choices of cue and stimulus representations—which were somewhat arbitrary. For this reason, we will focus our attention on the delay period in the remaining paper.

### Neuronal Coupling and Connectivity

**Figure 5:**
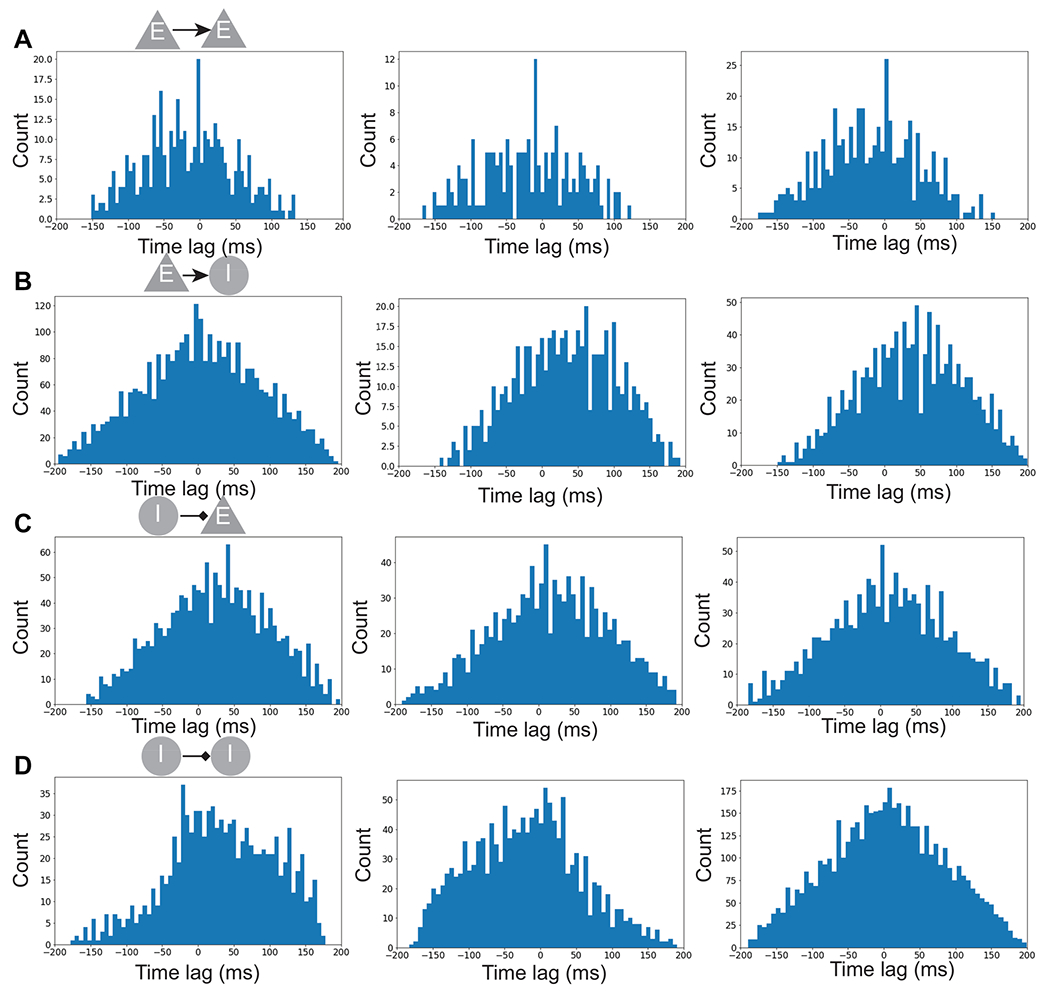
Coupling and cross-correlograms between rule-specific neurons. (A) Three cross-correlogram examples for E-E paired neurons. The trigger and target units are both rule-tuned neurons representing the same rule. (B) Three cross-correlogram examples of E-I paired neurons. The trigger unit is a rule-tuned neuron. (C) Three cross-correlogram examples of I-E paired neurons. The target unit is a rule-tuned neuron. (D) Three cross-correlogram examples of I-I paired neurons.

Next, we examined the pairwise neuronal coupling, especially those pairs with large (in absolute value) recurrent connection weights of the trained SRNN. In computing the spike-time cross-correlation between E-E, E-I, I-E and I-I neuronal pairs, we focused on the identified rule-tuned neurons as either the trigger or the target. Interestingly, we found many E-E pairs of rule-tuned neurons representing the same rule showed a high peak with a small time lag (2-8 ms) in their cross-correlograms (Fig. 5A). For E-I or I-E pairs, we often found a relatively large trough in their cross-correlograms (Fig. 5B,C). For I-I pairs, the cross-correlogram could have different profiles, depending on whether the rhythmically modulated units were the target (Fig. 5D, left panel), or trigger (Fig. 5D, middle panel), or both (Fig. 5D, right panel).

The investigation of cross-correlogram among simulated neurons may support the monosynaptic connections of task-modulated PFC neurons, and further provide new experimentally testable hypotheses. For instance, it has been shown that optogenetic mediodorsal (MD) thalamus activation can enhance cortical connectivity for enhancing the maintenance of working memory [19,37]. Investigation of the change in cross-correlograms may provide a testbed by computer simulations.

We further investigated whether single neuronal activities were related to any cluster within the trained network. For each excitatory neuron, we computed the trial variances at three different stages (cue period, delay period, and stimulus presentation period, each period with two rule conditions) and further embedded the 6-dimensional vector in a 2D space using *t*-distributed stochastic neighbor embedding (tSNE) algorithm [38]. Interestingly, we found functionally distinct clusters in the embedded space between rule-tuned and non-tuned excitatory populations (Fig. S4), yet no clear boundary between rule-specific subpopulations.

### Neural Trajectory Analysis

**Figure 6:**
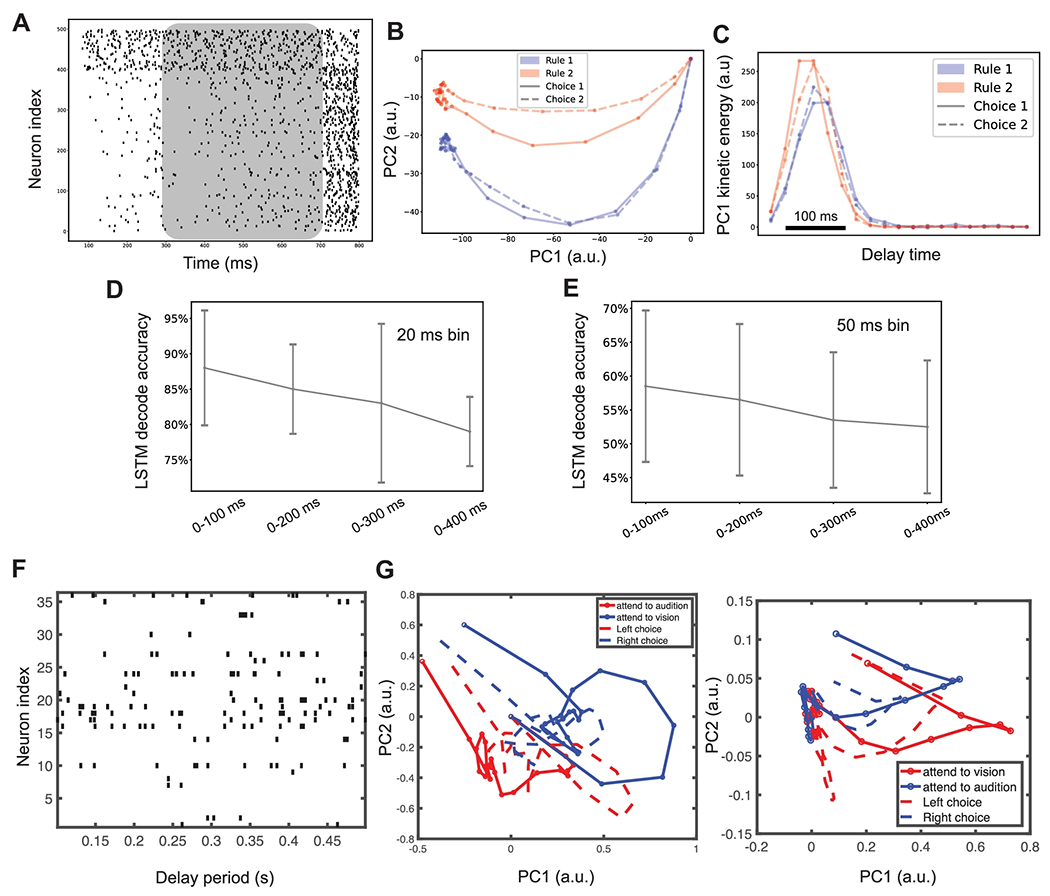
Neural trajectory analysis. (A) Spike rasters of 500 neurons during one single trial (cell #1-400 are excitatory neurons and cell #401-500 are inhibitory neurons). The shaded area denotes the delay period. (B) Neural trajectory **x**(*t*) of 400 excitatory neurons projected onto the PC1 and PC2 subspaces (from one trained SRNN) during the delay period. The rule-dependent information (blue vs. red) was maintained and propagated from the start (origin) to the end of delay period. Solid and dashed lines indicate the rule-dependent choices. (C) The curve of kinetic energy K(x) computed from PC1, which shows a high momentum in the first 100 ms, and then gradually decayed to very small value in the last 200 ms. (D) LSTM decoding accuracy (mean±SD, bin size: 20 ms) based on 15 dominant PCs of 400 excitatory neuronal firing activity during the delay period. (E) Similar to panel D, except for bin size of 50 ms. (F) One single correct trial of spike rasters of 36 mouse PFC neurons during 400-ms task delay. (G) Representative neural trajectories derived from experimentally collected mouse PFC neuronal spiking data during task delay. Results from two independent sessions were shown (first session: 36 PFC neurons, 40 trials; second session: 45 PFC neurons, 101 trials). Trial start was centered at the origin (0,0).

At the population level, the high-dimensional SRNN dynamics (Fig. 6A) during the delay period can be visualized via dimensionality reduction. Specifically, we conducted PCA and plotted the low-dimensional neural trajectory in the PC space (Fig. 6B). At the PC subspace, we observed the kinetic energy reached the maximum around 100 ms, and then decayed to around zero after 200 ms (Fig. 6C). Interestingly, our result was consistent with the primate PFC recording during working memory tasks [39], where the kinetic energy of population responses decayed after the cue onset and stayed low (inactive) in the state space. As a control, we also ran PCA based on the untrained SRNN or based on randomly shuffled recurrent weight matrix of the trained SRNN (Fig. S5); their results were qualitatively different from the neural trajectory derived from the trained SRNN.

Next, we ran an LSTM-based population decoding analysis based on 400 excitatory neuronal activities during the delay period. To test the rate code hypothesis, we used a 20-ms non-overlapping moving window to compute the trial-by-trial spike counts of individual neurons. We further projected them onto the PCA subspace and then decoded the rules from the 15 dominant PCs. We found out that 10-fold cross-validated (on 10% held-out trials) decoding accuracy saturated ~80-90% around 200 ms (Fig. 6D). The performance plateau can be explained by the flat temporal profile of kinetic energy in the last 150-200 ms of the delay period. These results also suggest that the additional rule information was more likely to be encoded by individual neurons’ spike timing at a finer timescale beyond the population firing rates. Changing the bin size from 20-ms to 50-ms resulted in degrade decoding accuracy and a similar trend (Fig. 6E).

Furthermore, as a comparison with experimentally collected data, we selected one recording session with 36 simultaneously recorded PFC neurons from a task-performing mouse (Fig. 6F). The PCA-derived neural trajectories are shown in Fig. 6G. Qualitatively, two separating trajectories that encoded two rules were noticed, but the trajectories derived from experimental data sessions were noisier (i.e., overlapping), possibly due to several factors: (i) the number of simultaneously recorded task-relevant. PFC units and the total unit yield; (ii) the number of successful trials in each recording session; and (iii) intrinsic experimental trial variability due to sources of other unobserved thalamocortical and modulatory input to the PFC.

### Error Trials Induced by Rule Encoding Uncertainties

**Figure 7:**
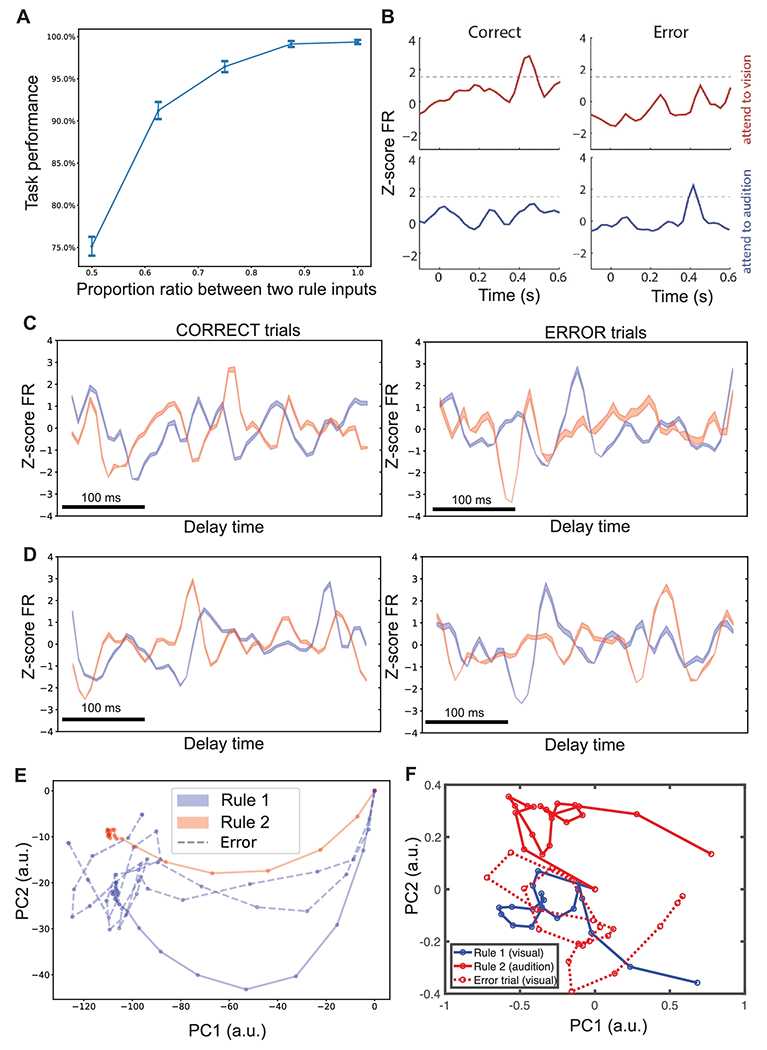
Error trials induced by rule-coding ambiguities. (A) Mean±SD performance curve of SRNN with respect to the mixed rule input with varying proportion ratios. (B) Example PSTHs of a neuron whose appropriate tuning of attending to vision rule was observed in error trials of attending to audition rule (Figure reproduced from (Schmitt et al., 2017) with permission). (C,D) Examples of comparative PSTHs of two rule-tuned units between correct and error trials. (E) Projection of two representative error trials (dashed lines, during the delay period) onto the PC subspace yielded neural trajectories deviating from the correct trials. The error-trial trajectories representing rule 1 gradually deviated from the blue trajectory and wandered around at the end of the delay period, creating a higher kinetic energy during the last 200 ms. (F) Neural trajectories derived from experimentally collected PFC neuronal spiking data in one recording session.

Thus far we have limited our analysis to correct trials performed by the trained SRNN. In animal experiments, behavioral errors may arise from executive error, sensory error, or both. The executive error is related to the rule encoding, whereas sensory error is related to the target cue perception. To emulate executive-type error trials, we introduced a mixed representation of two continuous rule inputs by varying their proportional ratios *q* and 1 – *q* (*q* proportion of rule signal 1 plus (1 – *q*) proportion of rule signal 2). We then converted the continuous input via the encoder into a discrete spike train. We fed the spike train that represented the mixed rule signal to the trained SRNN. As expected (Fig. 7A), the network produced an error trial with the highest probability when the ambiguity level was greatest (i.e., *q* = 0.5), and the average network performance gradually decreased when the degree of ambiguity increased.

Motivated from published experimental observations (Fig. 7B), we further examined the neural representations of single units during error trials. Generally, we found that the rule-specific tuned units during correct trials changed their firing profiles: (i) units tuned to rule 1 changed preferred tuning to rule 2, or vice versa (e.g., Fig. 7C); (ii) units tuned to both rules changed their peak-firing timing (e.g., Fig. 7D). At the population level, we also projected the neuronal responses of error trials onto the PC subspace; the error-trial trajectory showed a clear deviation from the correct-trial pattern. For instance, the trial mistakenly classified as rule 2 started a trajectory closer to rule 1, and then wandered in the neural state space before settling in a region closer to rule 2 (Fig. 7E). At the single-unit level, we only found very few examples (<5) that reversed their rule tunings in error trials (similar to experimental findings); this could be due to the sampling issue. As an additional comparison, we plotted the neural trajectories derived from experimentally collected PFC neuronal recordings (one session consisting of 32 simultaneously recorded neurons, 18 visual cue correct trials and 12 auditory cue correct trials). Qualitatively, the error trial trajectory displayed a distinct temporal pattern from the corresponding correct trial template and tended to be confused by the opposite rule (Fig. 7F).

### Impact of Elongated Delay Period

**Figure 8:**
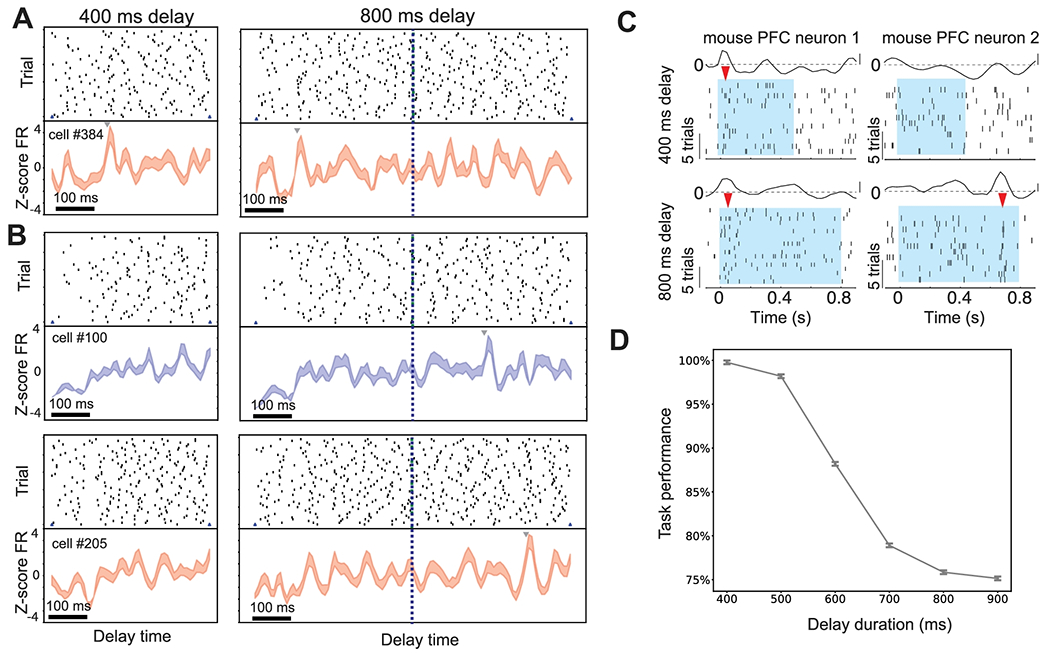
Neuronal activity during increased delay period. (A) One task-modulated neuron preserved the rule-specific peak timing in 800-ms as in 400-ms delay period. (B) New peak timing emerged in the late 800-ms delay period for two non-task-modulated neurons in the original setting. (C) Spike rasters and Z-scored PSTH examples of mouse PFC neurons during 400-ms and 800-ms delay periods. The vertical bar represents 2. Shaded areas denote the task delay (Figure reproduced from Schmitt et al., 2017, with permission). (D) Task performance degraded with increasing duration of task delay.

Motivated from the experimental manipulation [19], we further extended the 400-ms delay period to 800 ms in the testing phase, and ran the SRNN simulations for the complete 800-ms period. We further examined the neuronal responses in the elongated 400-ms period. Interestingly, we found that some non-task-modulated neurons developed a late peak in the 800-ms delay condition (Fig. 8B); in contrast, the task-modulated neurons that showed an early peak in the first 400-ms delay period preserved the peak timing even in the 800-ms delay (Fig. 8A). The latter case of neuronal responses implies the time-invariant temporal coding. Notably, our results produced qualitatively similar results as in some experimentally recorded PFC neurons from task-performing mice (Fig. 8C), where animals have been trained to perform the 2AFC task for both 400-ms and 800-ms task delay [19]. Furthermore, we observed a reduction in task performance with an increasing delay period (Fig. 8D), indicating the encoded rule information was gradually lost in working memory with increasing time. In total, we observed a small percentage (~.5%) of simulated units preserved their rule tunings in the new 800-ms delay period; and ~1.5% of simulated units showed emerged rule-tunings in the extended 400-800 ms period. In both cases, the mean firing rates of those units were not significantly different.

### Impact of Dropped Out Cortical Connections, Network Connectivity and E/I Balance

**Figure 9:**
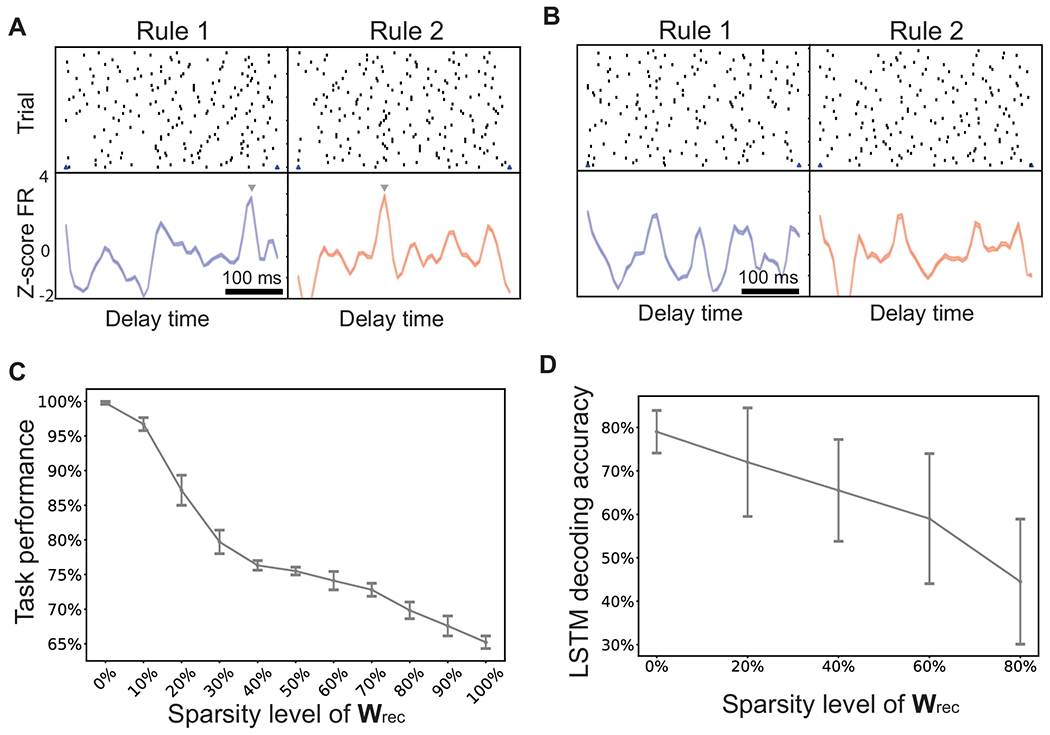
Impact of recurrent weight sparsification on neural representations and task performance. (A) Spike rasters and PSTHs of two task-modulated neurons during the delay period. Arrow marks the peak time. (B) The peak tuning of two neurons in panel A disappeared when ***W***^rec^ was sparsified. (C) Task performance degraded as a result of recurrent weight sparsification. Error bar denotes SD across 10 random realizations. (D) The cross-validated LSTM decoding accuracy (bin size: 20 ms) degraded with an increasing degree of ***W***^rec^ sparsity. Error bar denotes SD across 10 random realizations.

Furthermore, we investigated the fault tolerance of the SRNN with respect to the intracortical connectivity or recurrent weights. We randomly dropped out a small percentage of synaptic weights in ***W***^rec^ and set them to zeros. The network still performed well despite these lost synaptic connections. We ran the network simulations using the modified synaptic weight matrix and tested its impact on neural representations and task performance. We observed the sparsity affected the rule-specific tuning in excitatory neurons. Some observe peak timing disappeared with an increasing sparsity in ***W***^rec^ (Fig. 9A,B). With an increased sparsity level of recurrent weight matrix, we also observed a decrease in task performance (Fig. 9C) as well as cross-validated LSTM decoding accuracy (Fig. 9D). Notably, for the same level of sparsity, the task performance was more robust than the population decoding strategy during the delay period.

**Figure 10:**
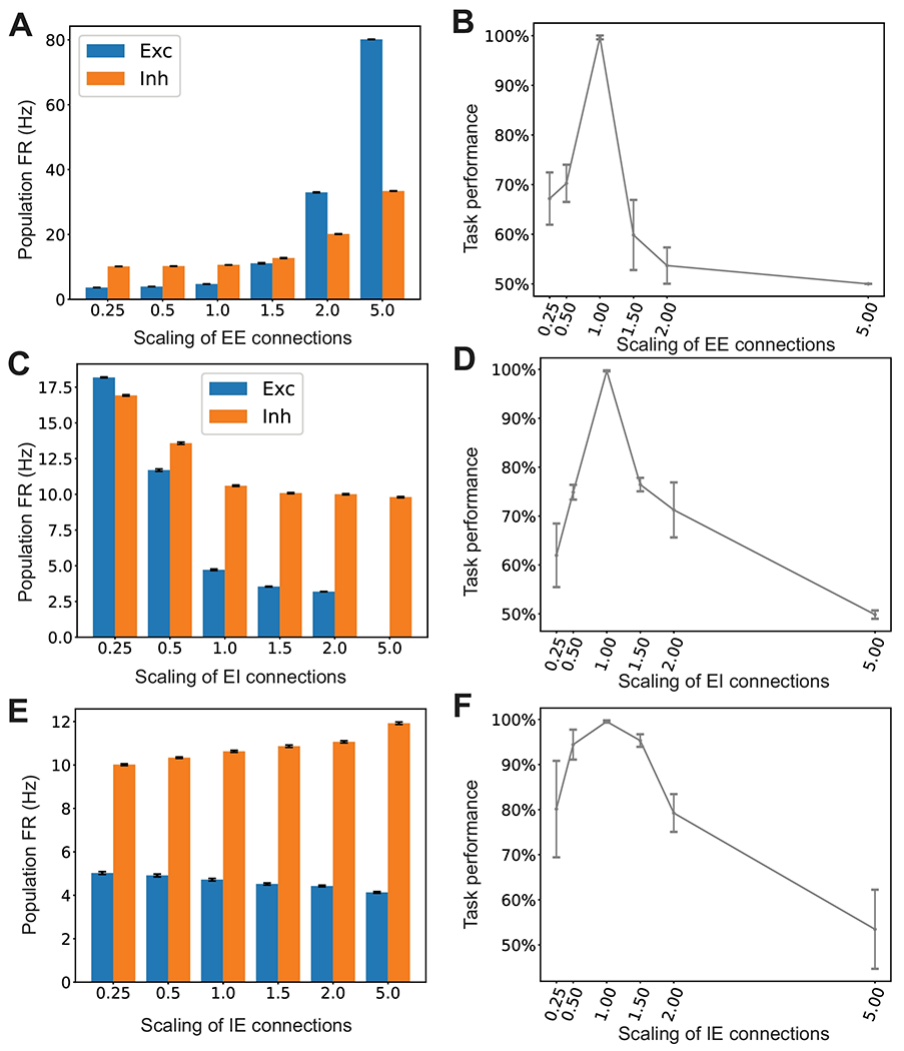
Impact of the cortical connectivity on the task performance and neural representations. (A) Population firing rates (FRs) of excitatory and inhibitory neurons changed with scaled EE connection strengths. Error bar denotes SEM. (B) Scaling up or down ***W***_EE_ changed the E/I level, and further affected the task performance. Error bar denotes the SD over 10 random realizations. (C,D) Similar to panels A and B, except for scaling ***W***_EI_ connection strengths. Note that in panel C, the E population firing rate is 0 for the scale of 5 (therefore invisible). (E,F) Similar to panels A and B, except for scaling ***W***_IE_ connection strengths.

To examine this effect of cell-type-specific neuronal connectivity on the neural representation and task performance, we scaled up or down the excitatory-to-excitatory ***W***_EE_ connectivity (by multiplying a scaling factor), and measured the changes in firing rates of E and I neuronal populations (Fig. 10A). As expected, the scaling factor affected the E/I firing rate and E/I balance. We further measured the impact on task performance in the trained SRNN (Fig. 10B). When the E/I was imbalanced, the task performance became poor. In the extreme condition where the overall excitation dominated over inhibition, the performance reduced to a chance level. As a comparison, we also scaled up or down the inhibitory-to-excitatory ***W***_EI_ or excitatory-to-inhibitory ***W***_IE_ connectivity and reported the firing rate changes (Fig. 10C,E) and task performance (Fig. 10D,F).

At the single-unit level, we found that the rule-tuned units decreased their peak firing rates according to the network E/I balance (Fig. S6). Specifically, we observed that the modified excitatory-to-excitatory or inhibitory-to-excitatory connections generated either conflicting or ambiguous rule tuning (Fig. S6A), or diminished rule tuning (Fig. S6B). The purpose of this investigation is to show how the impact of relative E/I imbalance may quantitatively affect the task performance and rule tuning. This became feasible by imposing Dale’s principle onto the SRNN. This result may make experimentally testable prediction. For instance, one can selectively activate or inactive PFC interneurons and quantify its impact on rule tunings. Notably, our computer simulation results were conceptually consistent with the reports shown in optogenetic PFC experiments [19].

### Extension and Generalization

To investigate the generalization ability of the SRNN, we adapted our computer experiments from the 2AFC task to the 4AFC task. Accordingly, we used four neuronal outputs to represent the four possible choices, and the decision was selected based on a softmax function. Since the computer task difficulty was increased, we thereby increased the size of the SRNN from *N*_rec_ = 500 to *N*_rec_ = 800. We noticed that the the final task performance of the trained SRNN slightly degraded (~95-98%); however, the rule-tuned neural representations were still preserved, at both the single unit and population levels (Fig. S7).

Additionally, we implemented a rate-based excitatory-inhibitory RNN model as described in [7] (results not shown). However, we could not reproduce the rule-specific sharp tuning (Fig. 3 and Fig. 4) and cross-correlogram (Fig. 5) at the single-cell level. Furthermore, we removed the constraint related to Dale’s principle and retrained the SRNN. Interestingly, we found that most of the reported phenomena still held, at both single cell and population levels (Fig. S8). Therefore, this result suggested that the emerging properties (rule-specific tuning and neural sequences) were not dependent on Dale’s principle.

### Experimental Predictions Based on Computer Simulations

Finally, computer modeling can also make experimentally testable predictions. Given the trained SRNN, we have shown that changing the network parameters or perturbing the recurrent weights can predict the impact on the task performance and neural representations (Fig. 10). We further made additional comments on hypothesis-driven prediction.

In light of PCA and neural trajectory analysis, we viewed the trained SRNN as a bistable attractor during the delay period in the 2AFC task, and the perturbation of neural trajectory during task delay pushed the decision region from one to the other in the neural subspace. To investigate the impact of signal-to-noise ratio (SNR) on the trained SRNN, we systematically varied the SNR by two ways: (i) varying the standard deviation (SD) or the background synaptic noise during the delay period; and (ii) introducing a 50-ms pulse distractor input during different windows of the delay period (0-100 ms, 100-200 ms, 200-300 ms, 300-400 ms). Accordingly, we reran the SRNN during testing and found the SRNN performance degraded with an increasing level of noise variance (Fig. S9A). Additionally, the performance decreased with an increasing level of distractor amplitude but showed less sensitivity during the late phase of task delay (Fig. S9B). This result supported that working memory may converge to a fixed point towards the end of the delay period, thereby being more tolerant to the distractor. These predictions can be tested in future rodent experiments.

## DISCUSSION

The SRNN models have recently become increasingly popular in modeling biological circuits [40–42]. In this work, we trained a SRNN model to replicate experimental spiking data of task-performing mice. While such RNN modeling effort was not new, our model imposed more biological constraints and incorporated state-of-the-art methods to improve the result interpretability. Overall, the SRNN provides a powerful and coherent framework for the neuroscience community and opens a new door to replicate existing and predict new experimental results due to the limitation of experimental conditions (e.g., insufficient unit yields or limited experimental trials). On the one hand, computational modeling may provide a data-driven approach to reproduce key experimental findings while tuning model parameters. On the other hand, computational modeling also offers a hypothesis-driven approach to make experimentally testable hypotheses by changing experimental conditions (e.g., trial duration), perturbing model parameters, and introducing external control simulations or disruption. Additionally, the SRNN can provide a means to model the LFP activity as well as spike-LFP interactions (e.g., spike-field coherence and spike-phase synchrony).

The context or rule-dependent, task behaviors have been modeled in the literature [2,7]. Mante *et al*. trained a rate-based RNN model of selection and integration to simulate the rule-dependent, computation in the monkey PFC [2]. The rate-based RNN model qualitatively reproduces the monkey’s behavior as well as the PFC population responses. There are several major distinctions between our model and theirs. First, our model is a SRNN, which uses a different training algorithm. Second, our model imposes biological constraints on the recurrent weight connections (i.e., Dale’s principle). Song *et al*. extended the rate-based RNN model by imposing similar biological constraints, but their learning framework was fundamentally different, from ours [7]. Notably, the distinction of excitatory and inhibitory neurons in the SRNN enabled us to examine the impact of cell-type-specific connectivity and E/I balance. Third, we introduced an additional biological constraint, known as SFA; this also turned out to be important, for training SRNN efficiently. Fourth, we imposed a firing rate regularization constraint, to encourage the sparsity of population responses.

Our modeling work was strongly motivated based on the previously published data [19]. Our central result is that the trained SRNN performing a 2AFC task can exhibit neural representations and dynamics that resemble the experimental findings of mouse PFC recordings. The resemblance between the SRNN and experimental data was manifested at the levels of single neurons and population responses. Although we didn’t model the neural sequence explicitly, the neural sequence representation emerged from the trained SRNN for performing the 2AFC task. Although the exact results may depend on the RNN’s hyperparameters (e.g., time constant, noise level, cost function and other simulation parameters), the general phenomena regarding rule-specific tunings and sequence representations are rather robust. Additionally, Dale’s principle is not a necessary condition for those emergent properties. Recent work [32] has suggested that both sequential and persistent activity are part of a spectrum that emerges naturally in the trained rate-based RNN under different conditions; and many factors, such as intrinsic circuit properties, temporal complexity of the task, Hebbian synaptic plasticity, delay duration variability, and structured dynamic inputs, can affect the neural representation of recurrent neurons in the trained network. Our SRNN modeling also confirmed similar findings. We found that the change in sparsity or strength of recurrent weight connections can affect neural representations under various test conditions. Furthermore, reverse engineering the RNN’s temporal dynamics can reveal low-dimensional neural attractor dynamics [9,24,33,43].

Our trained SRNN produced emergent beta oscillatory activity in approximately 25% of simulated inhibitory units, suggesting it may be difficult to observe in experimentally collected data due to low unit yields compared to computer simulations. Despite the low neuronal sampling issue, we have observed some evidence about beta and low-gamma (20-50 Hz) rhythmic activity at both spiking and LFP levels (Fig. 10E-G). Additionally, the beta/gamma rhythmic firing was consistent with the computational hypothesis in light of monkey PFC recordings during working memory tasks [44], despite the difference in the exact task. According to the dynamic coding hypothesis [36,45], the oscillatory activity is created by local feedback inhibition shared by local clusters of pyramidal neurons. During attractor activations, the recurrent, connections and specific synaptic potentiation produce a slight, excitatory bias in the subset of neuronal assemblies. Once they spike, they further activate a new wave of feedback inhibition and turn down the rest of the neurons. Beta oscillations are known to be more prominent in the absence of sensory drive (such as working memory delay) or motor movement (such as in motor preparation). Additionally, prefrontal beta oscillations have been linked to inhibition in executive control, as well as the formation of neural ensembles for top-down information [36]. Overall, our modeling study provides a computational framework to understand a number of PFC findings in cognitive control and working memory. For instance, we observed diverse (dynamic, persistent, and oscillatory) responses of single-unit activity during the delay period. The emerged oscillatory dynamics at the spiking activity of the SRNN demonstrate a clear computer modeling advantage at a fine temporal resolution, which is missing in the rate-based RNN models.

It is also worth pointing out several limitations of our modeling effort. First, the neural circuit for working memory and cognitive control is highly complex, yet we only focus on modeling the PFC circuit. However, it is well known that the PFC receives input from multiple brain regions, including the thalamocortical loop. Second, the exact, simulation results also depend on the choice of hyperparameters used in the SRNN (e.g., synaptic time constants). Additionally, the optimization and regularization may impact the simulation results. Third, we simply imposed an absolute refractory period for single spiking units in our computer simulations and did not explicitly model complex firing patterns such as bursting. Fourth, other unobserved processes, such as the reward-driven modulatory input and impulsive choices of mouse behavior, cannot be captured in the current model. Together, these reasons may possibly explain the difference in single-unit tuning curves between the simulated data and experimental data.

In general, RNN modeling provides a computational platform to investigate neuronal representations of brain circuits in a wide range of cognitive tasks. For instance, Yang *et al*. trained a rate-based RNN for performing 20 cognitive tasks that depend on working memory, decision-making tasks, categorization, and inhibitory control [8]. They found that after training, functionally distinct clusters emerged among recurrent neurons that were functionally specialized for different cognitive processes. Learning often gave rise to the compositionality of task representation. In addition, the trained network developed mixed task selectivity similar to the recorded PFC neurons after learning multiple tasks sequentially. Extension of our SRNN in a continual learning setting [8,46,47], will also be the subject of future investigation.

The SRNN can potentially be extended to modeling multi-area brain communications [48]. The PFC plays a key role in cognitive flexibility, and its neural representation and working memory function are highly dependent on its interaction with the mediodorsal thalamus (MD) [5,19,20,49]. Recently, the MD has been shown to play a modulatory role in cognitive control by augmenting effective connectivity between PFC neurons. In parallel to their experimental circuit dissection, computational models have been developed to account for thalamocortical or corticothalamic interactions [19,20,37]; extension of our SRNN framework to modeling the PFC-MD network with cell-type-specific projections and inter-area connection constraints will be the subject of our future investigation.

Finally, while our proposed SRNN is biologically plausible, its training procedure relies on supervised learning and back-propagation. However, it remains arguable whether the brain uses back-propagation to perform synaptic modification [50]. In contrast to the error-correcting learning mechanism, Hebbian plasticity and more specifically spike-timing-dependent plasticity (STDP), has become a well-established mechanism for learning. The STDP provides a biologically plausible unsupervised learning mechanism that locally modifies synaptic weights based on the degree of temporal correlations between the presynaptic and postsynaptic spike events [15,51–56]. Therefore, applying temporally asymmetric Hebbian learning for the SRNN to model neural sequences represents another important future research direction [57,58]. In our SRNN model, the hyperparameters of intrinsic properties (such as the membrane time constant, firing threshold, and reset potential) were fixed. Recently, it has been shown that joint optimization of synaptic weights (i.e., connectivity patterns) and membrane-related parameters (i.e., intrinsic neuronal properties) of the SRNN may help perform complex tasks that require information integration and working memory [41]. Furthermore, several state-of-the-art learning algorithms have been proposed for improving the optimization of SRNNs [59,60]. Future work will further improve the speed of SRNN training and validate it in a realistic and complex task setting.

## Supplementary Material

1783417_Sup_Material

## Figures and Tables

**Table 1:** A summary of symbols and notations in SRNN.

Symbol	Description

*S*^cueing^ (*t*)	Cueing input spike for SRNN time step *t*
*S*^sensory^ (*t*)	Sensory input spike for SRNN time step *t*
*S*^rec^ (*t*)	Recurrent spike for SRNN at time step *t*
***W*** ^rec^	Recurrent connection weights of SRNN
***W*** ^out^	Connection weights of the readout layer
***W*** ^cueing^	Connection weights of the cueing input layer
***W*** ^sensory^	Connection weights of the sensory input layer
Θ(·)	Heaviside step function
*σ*(·)	Fast sigmoid function
⊙(·)	Elementwise multiplication
*ϕ* ^exc^	The ratio of excitatory neurons
*v*_rest_	Voltage constant at the resting state
*v*_th_	Base voltage threshold constant for firing spike
*τ*_m_	Time constant of membrane potential
*τ*_syn_	Time constant of synapse
*τ*_a_	Time constant of spike frequency adaptation (SFA)
*τ*_ref_	Time constant for the refractory period
*η*	Learning rate
*g*	Rescaling constant for ***W***^rec^
*β*	Scaling constant for the surrogate gradient
*ψ*	Scaling constant for spike frequency adaptation (SFA)

**Table 2:** Hyperparameters used in the SRNN.

Symbol	Description

*I*^base^(*t* = 0)	$0.041\times N\left(\mu =2,\sigma^{2}=0.01\right)$, truncated in [1.8, 2.4]
*ξ*^base^(*t*)	$N\left(\mu =0,\sigma^{2}=4\times 10^{-6}\right)$
*ξ*^*m*^(*t*)	$N\left(\mu =0,\sigma^{2}=16\right)$
*v*_rest_	−65 mV
*v*_th_	−50 mV
*ϕ*_exc_	80%
*dt*	2 ms
*β*	1000
*R*	10^−2^ Gigaohm
*τ*_mem_	20 ms
*τ*_syn_	35 ms for excitatory neurons, 40 ms for inhibitory neurons
*τ*_a_	400 ms
*τ*_ref_	6 ms
*η*	3 × 10^−4^
*g*	1.5
*ψ*	1.6
λ	10^−9^

**Table 3:** Parameters used in the encoder and decoder.

Symbol	Description

*V*^encoder^(*t* = 0)	0.0 mV
*V*^decoder^(*t* = 0)	0.0 mV
$v_{\text{rest}}^{\text{encoder}}$	0.0 mV
$v_{\text{rest}}^{\text{decoder}}$	0.0 mV
$v_{\text{th}}^{\text{encoder}}$	1.0 mV
$v_{\text{th}}^{\text{decoder}}$	1.0 mV
*I*^encoder^(*t* = 0)	0.0 mA
*I*^decoder^(*t* = 0)	0.0 mA
*R* ^encoder^	1 Ohm
*R* ^decoder^	1 Ohm
$\tau_{\text{m}}^{\text{encoder}}$	5 ms
$\tau_{\text{m}}^{\text{decoder}}$	5 ms
$\tau_{\text{syn}}^{\text{decoder}}$	20 ms

## Data Availability

All custom computer software files are available at https://github.com/Jakexxh/srnn-2afc.
