# Supplementary material for: Spiking Recurrent Neural Networks Represent Task-Relevant Neural Sequences in Rule-Dependent Computation
Source: Cognit Comput. Author manuscript; Available in PMC 2024 Jul 1. (PMC10530699; doi:10.1007/s12559-022-09994-2)
Supplement: 1783417_Sup_Material [file NIHMS1783417-supplement-1783417_Sup_Material.pdf]

Supporting Information

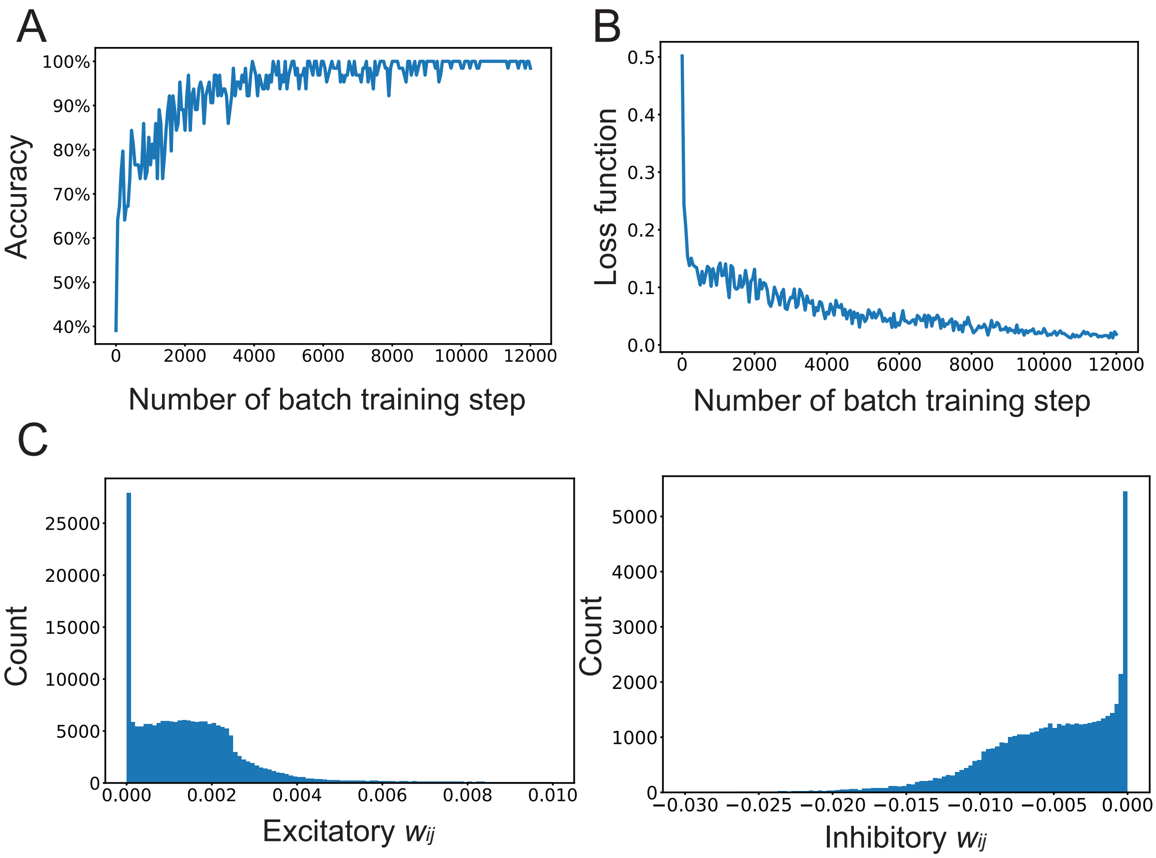

Figure S1: **SRNN Learning.** (**A**) Learning curve for the classification accuracy. (**B**) Learning curve for the regularized loss function. (**C**) Distributions of learned excitatory and inhibitory synaptic connections of a trained SRNN.

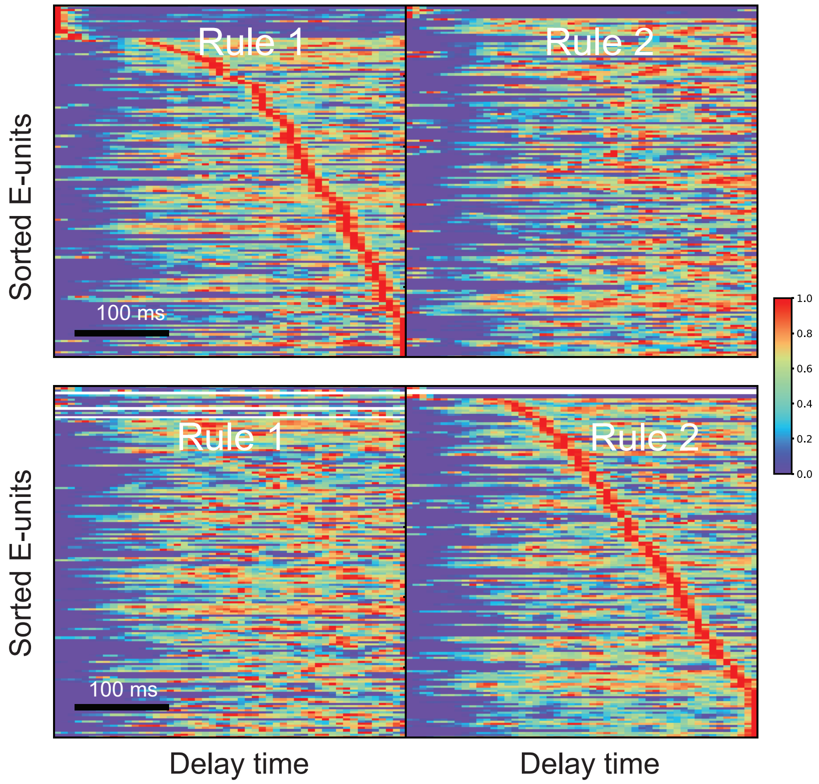

Figure S2: **Rule-specific neural sequential activity.** Heat maps of normalized mean firing rates (each row was normalized between 0 and 1) of all excitatory neurons from one trained SRNN.

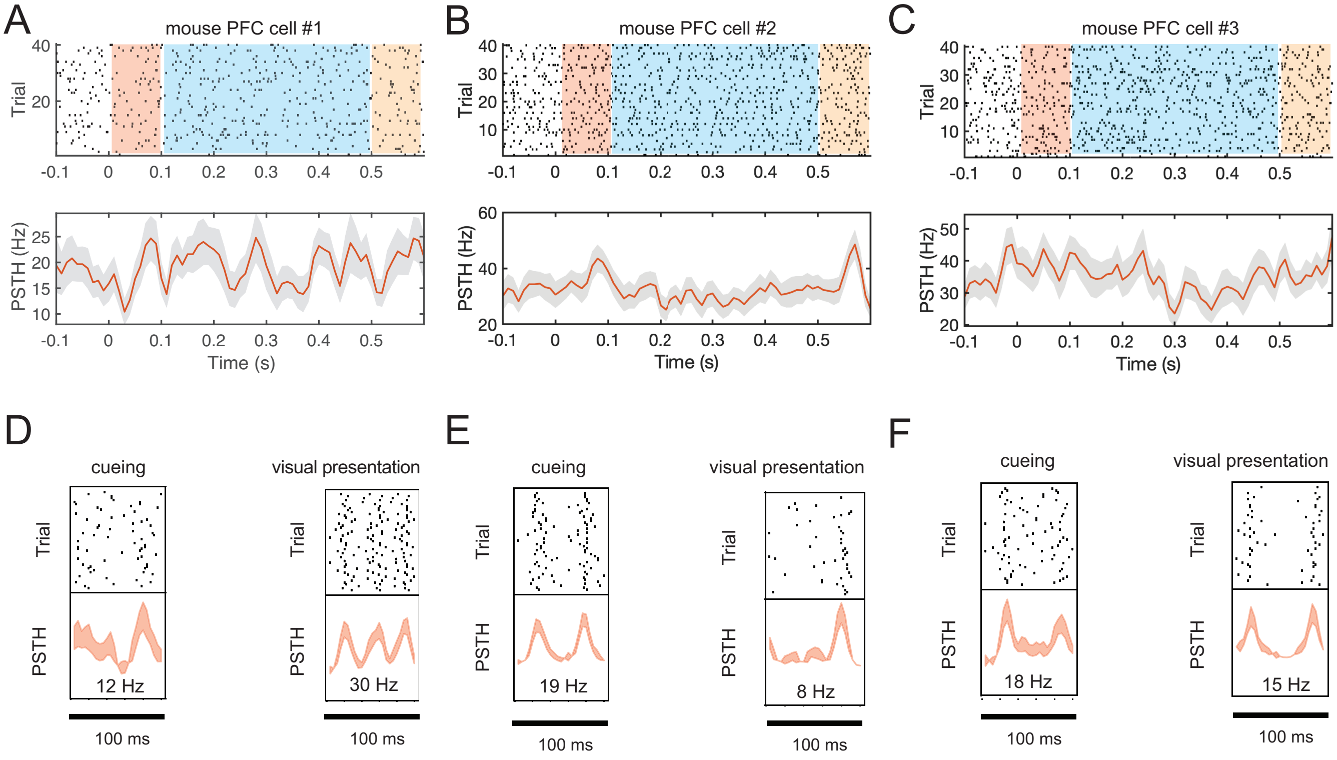

Figure S3: **Experimental PFC recordings from task-performing mice (Schmitt et al., 2017).** (A-C) Examples of three PFC single units during the cueing period (0-0.1 s), delay period (0.1-0.5 s), and visual stimulus presentation (0.5-0.6 s) in the 2AFC task. (D-F) Examples of simulated SRNN units during 100-ms cueing and 100-ms stimulus presentation periods. Only one cue and visual stimulus presentation are shown for the purpose of illustration. Mean firing rates are shown in the panel.

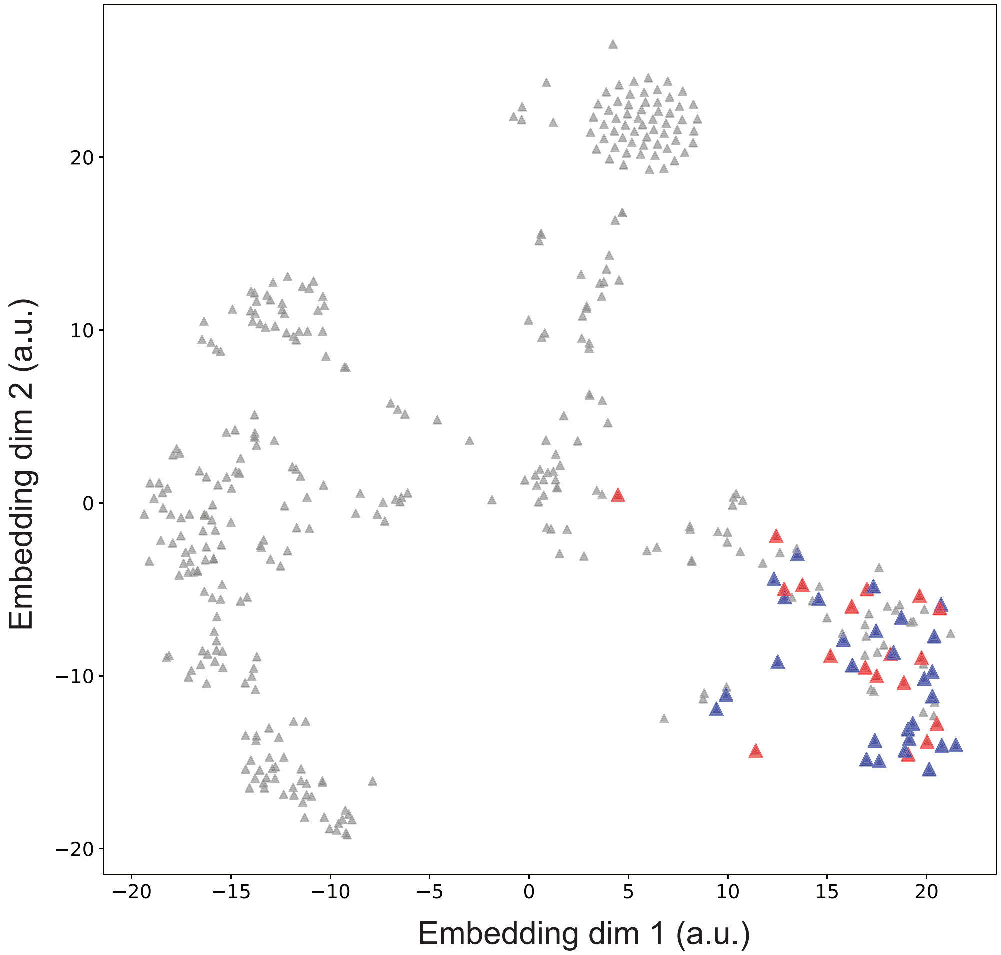

Figure S4: Visualization of functional clusters via stochastic neighbor embedding (tSNE). Each triangle denotes one excitatory neuron, but rule-tuned neurons are shown with bigger markers. Red triangles denote rule 1 tuned excitatory neurons, and blue triangles denote rule 2 tuned excitatory neurons.

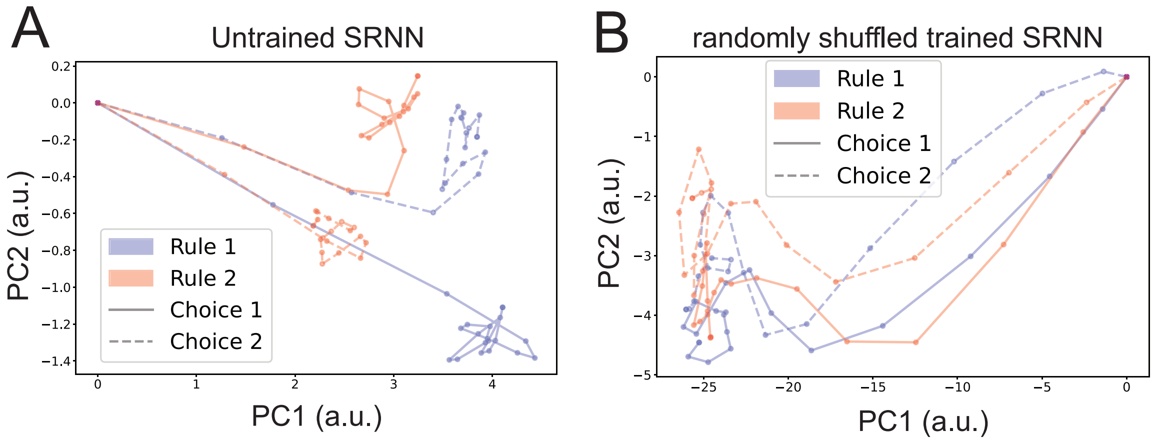

Figure S5: **Neural trajectory in subspace.** Control experiments of PCA-derived neural trajectories based on the untrained SRNN (panel A) and randomly shuffled recurrent weight matrix of the trained SRNN (panel B). These neural trajectories appeared erroneously mixed in color compared to Fig. 6B.

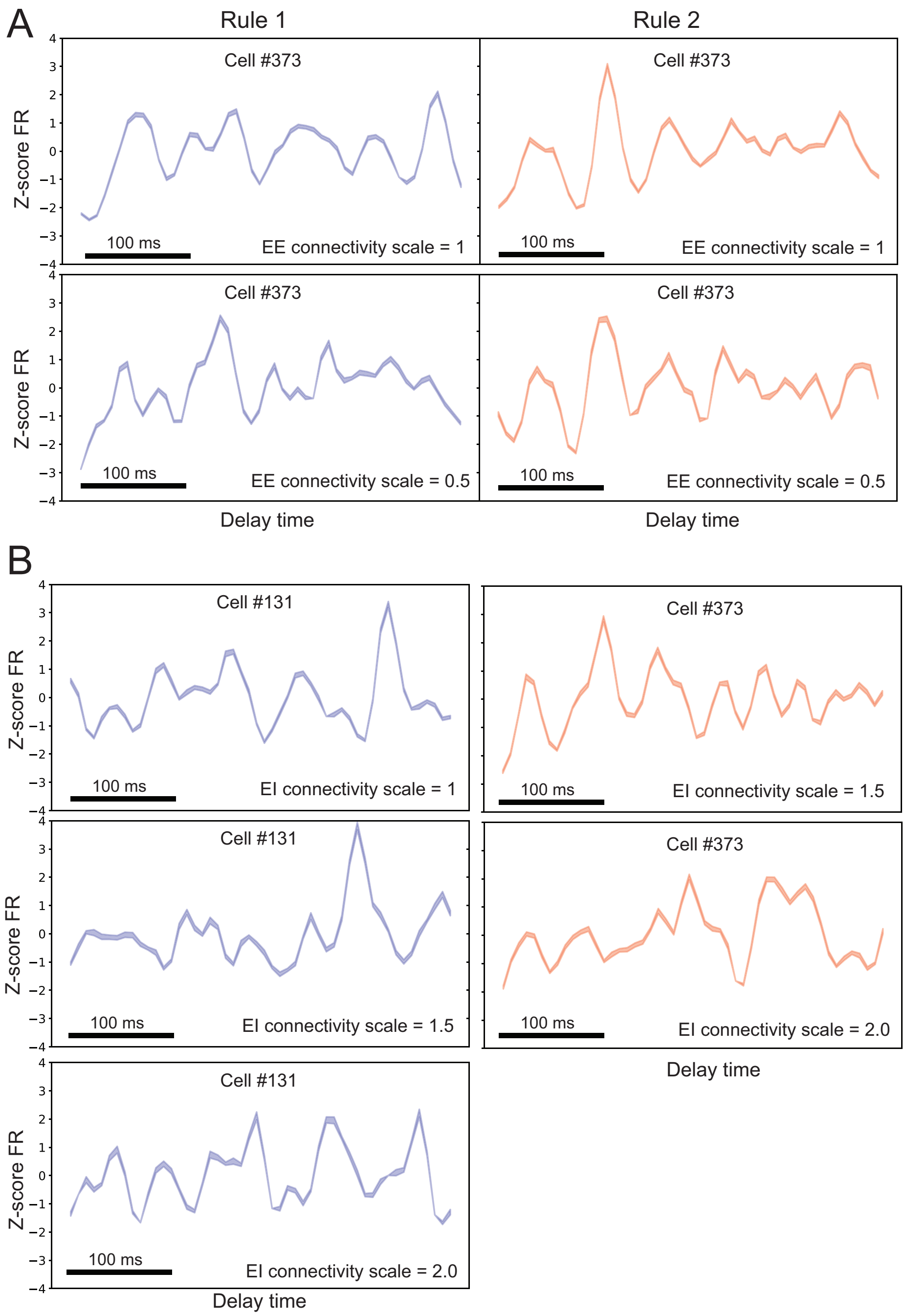

Figure S6: **Change in rule-specific tuning due to modified EE or EI synaptic connectivity**. (A) Modification of EE connectivity (scaling of 0.5) generated inappropriate tuning peaks at the opposite rule or reduced the peak tuning. (B) Modification of EI connectivity (by scaling of 1.5 and 2.0) changed or diminished the peak tuning.

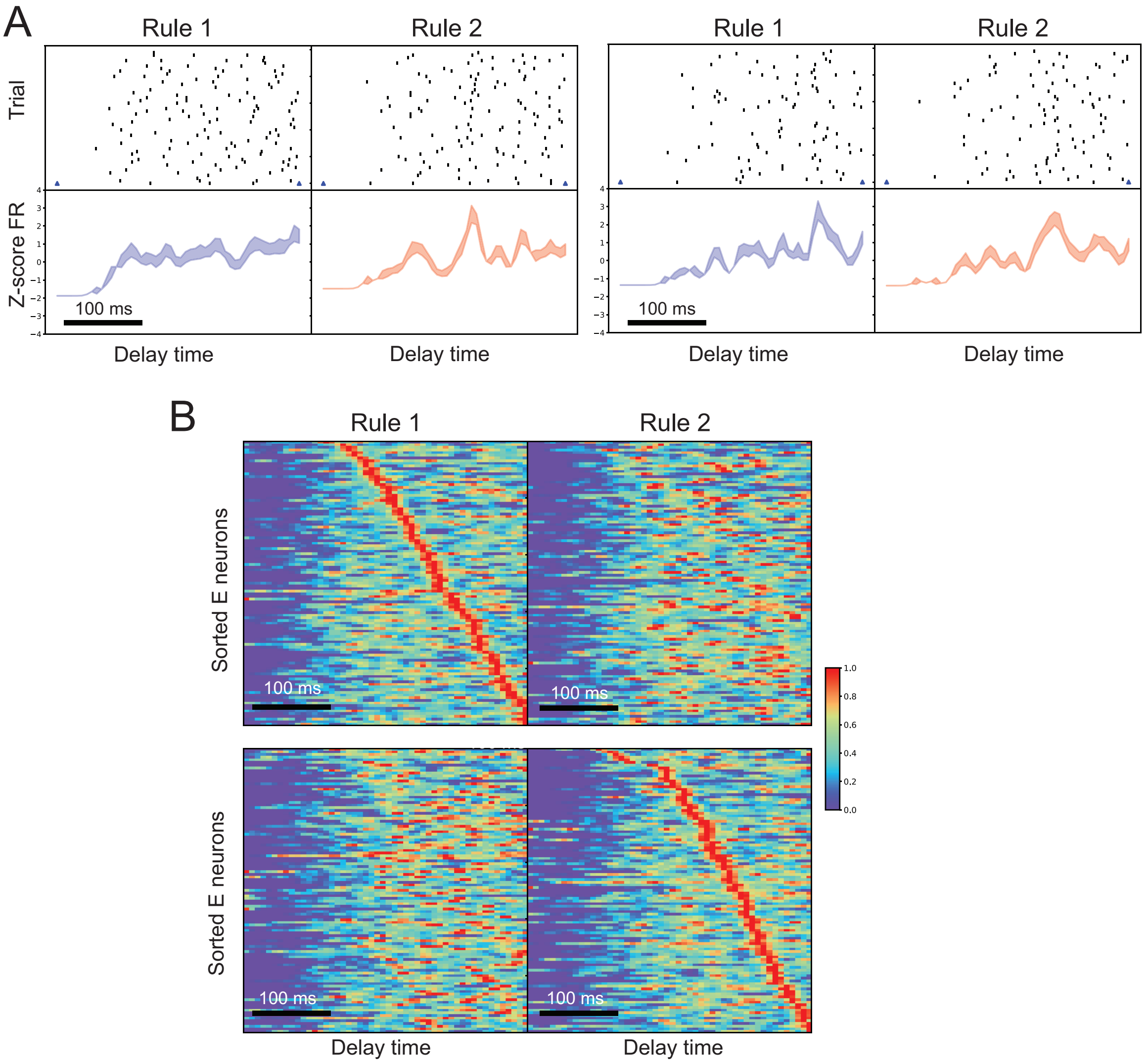

Figure S7: **Rule-specific tuning and neural sequence were preserved during the delay period of the 4AFC task.** (A) Spike rasters and PSTHs of two representative rule-tuned excitatory neurons. Shaded area denotes SEM. (B) Neural sequential activity formed by rule-tuned excitatory neurons.

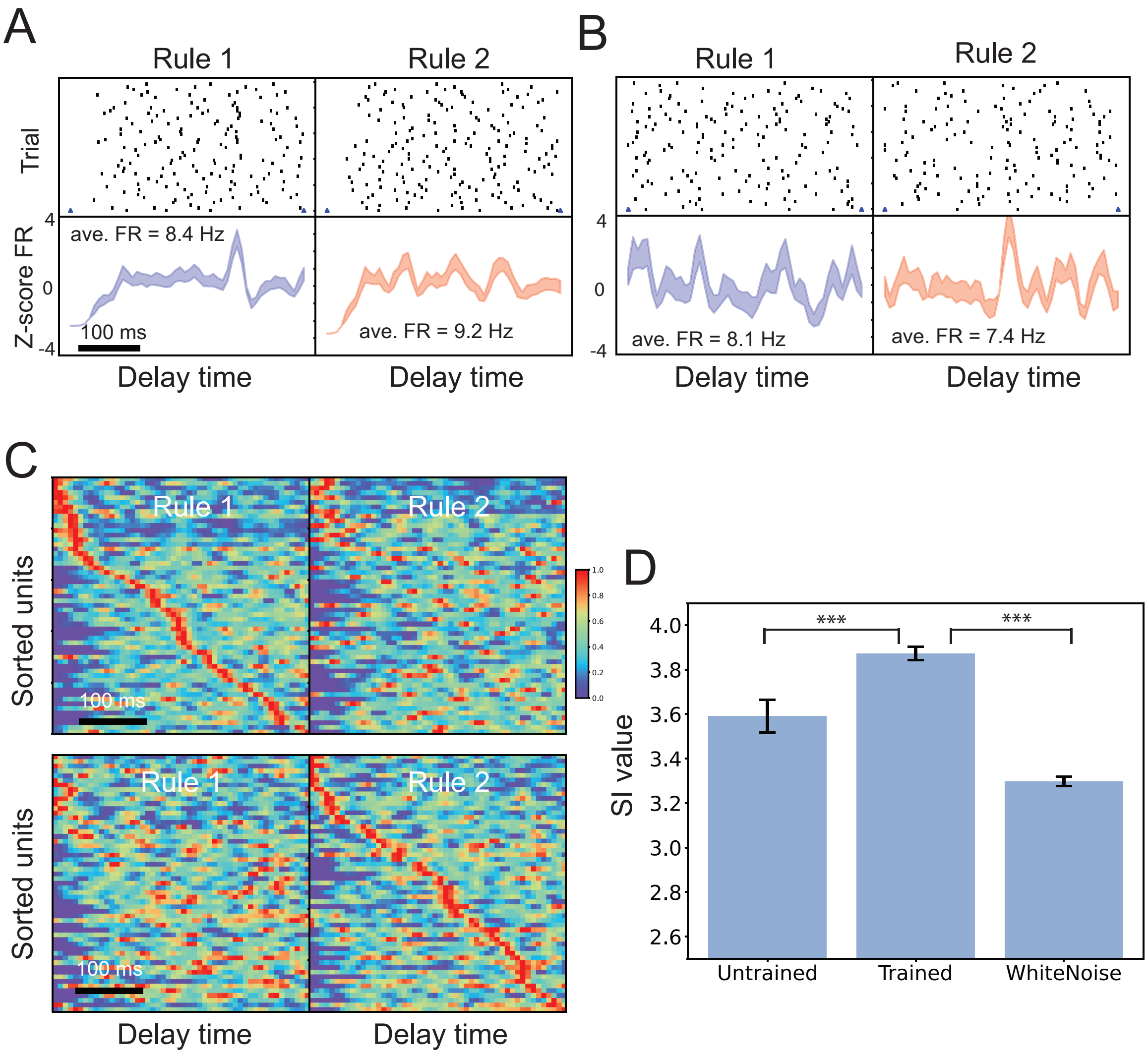

Figure S8: **Rule-specific tuning and neural sequence were preserved during the delay period of the 2AFC task, despite the relaxation of Dale's principle in the SRNN.** (A,B) Spike rasters and PSTHs of two representative rule-tuned neurons. Shaded area denotes SEM. Mean firing rate (FR) is marked along the PSTH. (C) Neural sequence formed by rule-specific excitatory neurons. (D) Comparison of SI statistics between three different conditions. Mean±SD statistics were computed from 10 untrained and 10 trained SRNNs. ***, $p < 10^{-3}$.

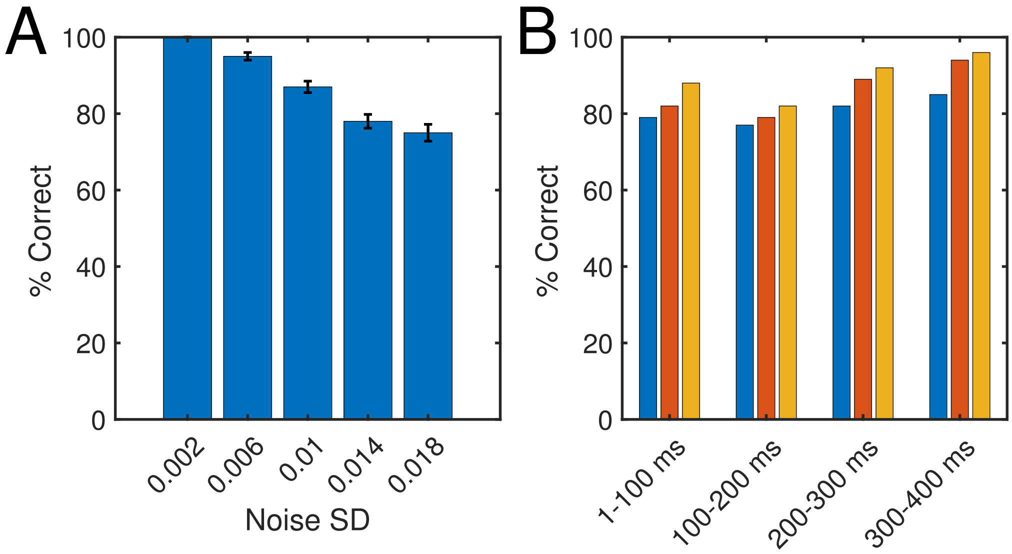

Figure S9: **Impact of SNR and distractor during the delay period on task performance.** (A) Task performance of SRNN decreased with increased level of noise or reduced SNR (error bar denotes SEM from $n = 10$ Monte Carlo simulations). (B) Task performance of SRNN varied with respect to different distractor timing and amplitude (blue: high; red: medium; green: low amplitude) during 400-ms delay period.
